# Applying polygenic risk score methods to pharmacogenomics GWAS: challenges and opportunities

**DOI:** 10.1093/bib/bbad470

**Published:** 2023-12-27

**Authors:** Song Zhai, Devan V Mehrotra, Judong Shen

**Affiliations:** Biostatistics and Research Decision Sciences, Merck & Co., Inc., Rahway, NJ 07065, USA; Biostatistics and Research Decision Sciences, Merck & Co., Inc., North Wales, PA 19454, USA; Biostatistics and Research Decision Sciences, Merck & Co., Inc., Rahway, NJ 07065, USA

**Keywords:** polygenic risk score, pharmacogenomics GWAS, challenges and opportunities, Eurocentric bias, Bayesian, multiple traits

## Abstract

Polygenic risk scores (PRSs) have emerged as promising tools for the prediction of human diseases and complex traits in disease genome-wide association studies (GWAS). Applying PRSs to pharmacogenomics (PGx) studies has begun to show great potential for improving patient stratification and drug response prediction. However, there are unique challenges that arise when applying PRSs to PGx GWAS beyond those typically encountered in disease GWAS (e.g. Eurocentric or trans-ethnic bias). These challenges include: (i) the lack of knowledge about whether PGx or disease GWAS/variants should be used in the base cohort (BC); (ii) the small sample sizes in PGx GWAS with corresponding low power and (iii) the more complex PRS statistical modeling required for handling both prognostic and predictive effects simultaneously. To gain insights in this landscape about the general trends, challenges and possible solutions, we first conduct a systematic review of both PRS applications and PRS method development in PGx GWAS. To further address the challenges, we propose (i) a novel PRS application strategy by leveraging both PGx and disease GWAS summary statistics in the BC for PRS construction and (ii) a new Bayesian method (PRS-PGx-Bayesx) to reduce Eurocentric or cross-population PRS prediction bias. Extensive simulations are conducted to demonstrate their advantages over existing PRS methods applied in PGx GWAS. Our systematic review and methodology research work not only highlights current gaps and key considerations while applying PRS methods to PGx GWAS, but also provides possible solutions for better PGx PRS applications and future research.

## INTRODUCTION

Polygenic risk scores (PRSs) have recently emerged as promising tools in disease genome-wide association studies (GWAS) for predicting human diseases and complex traits. This is particularly important for diseases/complex traits with polygenic genetic architectures, where many genetic variants have small but genuine effects that do not reach the genome-wide significant threshold [1]. A PRS combines multiple single nucleotide polymorphisms (SNPs) into a single aggregated score that can be used to predict disease risk. It is an individual-level score calculated based on the number of risk variants that a person carries, weighted by SNP effect sizes that are derived from an independent large-scale discovery GWAS. Thus, this score represents the total genetic risk of a specific individual for a particular trait, which can be used for clinical prediction or screening. To date, many PRSs have been successfully used in disease risk prediction and population stratification. For example, Khera *et al*. [2] developed a PRS for coronary artery disease (CAD), where the PRS-high group (i.e. the top 8.0% of the population) inherited $\ge$ 3-fold increased risk for CAD. Mavaddat *et al*. [3] built a PRS that was optimized for predicting estrogen receptor (ER)-specific disease. Individuals with the highest 1% of PRS had 4.37-fold (increased) risk of developing ER-positive disease, whereas those with the lowest 1% of PRS had 0.16-fold (decreased) risks.

In contrast to disease genetic studies, pharmacogenomics (PGx) studies explore how genetic variation influences drug responses, including drug metabolism, efficacy and toxicity, with the ultimate goal of improving and personalizing drug therapy. Such influence is usually via alterations in a drug’s pharmacokinetics (PK, i.e. absorption, distribution, metabolism, elimination) or via modulation of a drug’s pharmacodynamics (PD, i.e. modifying a drug’s target or perturbing biological pathways that alter sensitivity to the drug’s pharmacological effects) [4]. Like many complex traits, most drug responses in PGx are extremely polygenic [5, 6, 7]. For example, Muhammad *et al*. showed that the six PD and five PK phenotypes they studied were highly heritable, and the majority of the heritability was explained by small-effect and moderate-effect variants instead of large-effect variants, which demonstrates the potential for using PRS approaches in the clinic to improve prediction of PD/PK phenotypes to fulfill the promise of precision medicine [7]. There are some emerging PRS applications in PGx studies published in most recent years. Zhang *et al*. [8] developed a PRS from schizophrenia GWAS summary statistics reported by the Psychiatric Genomics Consortium for schizophrenia risk and showed that patients with higher PRSs tended to have less improvement with antipsychotic drug treatment. Similarly, Damask *et al*. [9] found that both the absolute and relative reduction rates of major adverse cardiovascular events by alirocumab treatment compared with placebo were greater in patients with higher PRS in the ODYSSEY OUTCOMES Trial, where they constructed the PRS using disease GWAS summary statistics from a genome-wide meta-analysis of CAD risk. In addition, Marston *et al*. [10] used a PRS constructed from 27 SNPs derived from a recent large-scale disease GWAS study (a meta-analysis GWAS for CAD) to successfully predict benefit from Evolocumab therapy in patients with atherosclerotic disease. On the other hand, instead of building PRSs with disease GWAS data, a few published studies choose to construct PRSs from drug-related data for safety or efficacy drug response predictions. For example, Lanfear *et al*. [11] built an efficacy PGx PRS from a PGx genome-wide analysis of $\mathrm{\beta}$-blocker $\times$ SNP interaction, and successfully predicted all-cause mortality ($\mathrm{\beta}$-blocker benefit) in the European population. These examples highlight the potential benefits of developing PRSs for (safety or efficacy) drug response predictions in PGx studies, as well as their potential utilities in clinic.

PRS applications in PGx studies have been reviewed by several papers with different focuses. Specifically, Johnson *et al*. [12] and Cross *et al*. [13] reviewed 51 and 63 PRS application papers, respectively, focusing on the PRSs built with variants from disease GWAS. They included an overview of the PRS applications and successful findings in different disease areas, challenges and reporting guidelines. Siemens *et al*. [14] reviewed 89 papers, with a focus on the PRSs derived from pharmacogenetic variants associated with drug responses in either candidate gene PGx studies or PGx GWAS. In addition, the authors also reviewed the strategies of PRS performance evaluation and validation. Similarly, Kumuthini *et al*. [15] focused on the validation of the PRSs in PGx, and the potential impact on their translation into clinical utility. Regarding the PRS methods developed in the disease genetics field, there are several papers reviewing the PRS methodologies using disease GWAS summary statistics, focusing on the genotype (main or prognostic) effects only [16, 17, 18]. In contrast, PRSs built for PGx studies from randomized clinical trials (RCTs) need to handle both prognostic and predictive effects. This is because in PGx studies, a patient’s clinical outcomes are influenced by both prognostic and predictive factors. A prognostic biomarker provides information about an endpoint (i.e. clinical outcome) irrespective of the treatment type. However, a predictive biomarker is associated with an endpoint related to the treatment type (i.e. predicting treatment benefits). In the PRS context, the prognostic effect measures the main genotype (G) association strength with clinical outcome before any treatment intervention. On the other hand, the predictive effect measures the G x T interaction association strength with clinical outcome after treatment. While current practice involves in directly applying disease GWAS-based PRSs to PGx studies, Zhai *et al*. [19] pointed out that this approach might not fully recover the heritability of drug response since it relied on a stringent assumption which was barely satisfied in real PGx data. The authors further proposed a series of PRS-PGx methods using PGx GWAS summary statistics instead.

PRS modeling in PGx GWAS shares the same challenges as those in disease GWAS. These challenges include trans-ethnic bias across populations (i.e. GWAS sample size for non-European populations is relatively lower and a PRS derived from one population is expected to perform less well in other populations due to differences in allele frequencies, linkage disequilibrium (LD) patterns and effect sizes across different populations) [20, 21, 22] and architectural diversity across multiple correlated traits [23]. Some other challenges include the lack of clear guidelines on clinical interpretation of PGx polygenic models, PGx studies usually requiring more well-defined drug response clinical endpoints, and the lack of PRS reporting guidelines. Despite previous discussions and summaries of these challenges (for example, in the above review papers), possible strategies and solutions to tackling these challenges remain unclear. Therefore, it is critical to take one step further by proposing new PRS application strategies and methods. To gain insights into this landscape, we first conduct a systematic review of the current progress in terms of both the PRS applications and statistical methods development in PGx GWAS. We identify 90 papers published by 11 March 2022 for our PRS PGx applications review, and summarize 23 PRS methods used in these papers in our PRS methods review. We further analyze these systematic review results to provide insights into the status, trends and challenges of PRS applications and method developments in PGx GWAS and discuss potential areas for improvement.

Compared with PRS modeling in disease GWAS, PRS analysis in PGx GWAS with drug response endpoints (efficacy or safety) is more challenging and faces additionally unique challenges. They include the lack of knowledge about whether to use PGx GWAS, disease GWAS or both GWAS/variants in the base cohort (BC) for PRS construction, the small sample sizes in PGx GWAS from RCTs (compared to large disease cohorts) which often result in low power for prediction or association analysis, and the more complex statistical modeling for handling both prognostic and predictive effects simultaneously. There is a trade-off between choosing PGx and disease GWAS (summary statistics) data in the BC to build PRSs. Choosing disease GWAS data, which typically has a large sample size, usually provides large power for prognostic effect prediction, but low power for predictive effect (i.e. genotype-by-treatment interaction) prediction. In contrast, choosing PGx GWAS data, which typically has a relatively small sample size, usually provides lower power for prognostic effect prediction, but likely larger power for predictive effect prediction since PGx variants used for PRS construction are directly drug response related.

In this paper, rather than choosing either PGx or disease GWAS alone in the BC to build a PRS, we propose a new strategy to leverage both disease and PGx GWAS summary statistics. This approach benefits from the large sample size in disease genetic studies and the additionally strong predictive effects in PGx studies. In addition, similar to the cross-population disease GWAS PRS, leveraging and properly modeling trans-ethnic populations can potentially reduce the prediction bias for cross-population prediction in PGx GWAS. To overcome the challenge of Eurocentric or trans-ethnic bias in PGx GWAS, we extend the PRS-PGx-Bayes method [19] to conduct the cross-population PRS modeling with shared shrinkage parameters among multiple populations. The new method simultaneously handles both prognostic and predictive effects. We further perform extensive simulation studies to compare our novel PRS methods using both internal validation with a cross-validation strategy and external validation with an independently simulated validation dataset, as suggested by Siemens *et al*. [14] and Kumuthini *et al*. [15]. Furthermore, integrating multiple genetically correlated traits can potentially increase the effective sample size in PGx GWAS, and thus improve the power of PRS association test, PRS prediction accuracy and PRS-based patient stratification. To investigate the impact of complex genetic architectures among multiple traits, Zhai *et al*. [24] systematically reviewed and compared multiple types of multi-trait PRS methods including regression-based methods (mtPRS-ML and mtPRS-MR), meta−/multi-GWAS-based methods (mtPRS-minP and mtPRS-GSEM), PCA-based method (mtPRS-PCA), and omnibus mtPRS method (mtPRS-O) under various genetic architectures. In this paper, we briefly summarize the current status of applying mtPRS methods to PGx GWAS and main observations from our previous mtPRS method research work [24].

In summary, in this paper, we aim to provide an in-depth overview of the current status of both PRS applications in PGx GWAS and the PRS methods used in those applications, identify the main gaps and challenges, and then propose the possible solutions including two new PRS application strategies and methods for filling the gaps and tackling the challenges. The overall workflow of the paper is summarized in Figure S1.

## METHODS

Our review aimed to summarize the current applications of PRSs in PGx GWAS as well as the PRS analysis methods used in the applications. The detailed paper searching workflow is provided in Figure S2. The study screening was conducted by two independent reviewers (Z.S. and S.J.) to minimize selection bias. In the ‘Identification’ step, we used the same search terms as used by Johnson *et al*. [12], which mainly included two categories: polygenic score related terms and drug response related terms. Our paper search was based on Medline and EMBASE databases up to 11 March 2022, separately. Non-English publications were excluded due to resource constraints. In the ‘Pre-screening’ step, we excluded publications by title and abstract. Publications with abstract only, reviews, letters, notes, etc. were excluded as they did not contain enough details about PRSs [e.g. base and target cohorts (TC)] for our review. The remaining articles that were not drug related and did not use qualified PRSs (e.g. not genome-wide, not genetic variation based or unweighted PRSs) were further excluded. In the ‘Combination’ step, we combined the papers from Medline and EMBASE together and removed duplicate records. In the ‘Screening’ step, records were further excluded based on a full-text screen due to either unqualified publication types or unqualified approaches to build PRSs. Specifically, papers that were not drug related, not drug response PGx study related, not constructing qualified PRSs or only working on TC via cross-validation, were excluded. The whole workflow followed the Preferred Reporting Items for Systematic Reviews and Meta-Analysis (PRISMA) guidelines [25] to ensure completeness of the review.

In addition to summarizing the PRS applications in PGx studies, we also summarized the PRS analysis methods used in the 90 identified PGx PRS application papers as well as some additional PRS methods reviewed in other methodology review papers [16, 17, 18]. Regarding the challenges of applying PRSs to PGx GWAS, in this paper we mainly focus on three key challenges: (i) the lack of knowledge about whether to choose PGx, disease or a combination of both PGx and disease GWAS summary statistics in the BC for PRS construction; (ii) the Eurocentric or trans-ethnic bias in cross-population PRS prediction and (iii) the small sample size, low power and more complex PRS modeling in PGx GWAS. We propose two novel PRS strategies and methods to overcome the challenges, which provide potential solutions for better future PRS applications in the field.

## RESULTS

### Overview of main challenges of PRS applications in PGx GWAS

**Figure 1:**
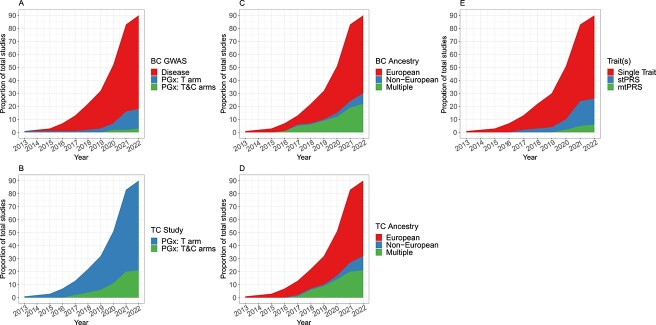
Number of PRS applications included in the review over time. (**A**) Source types of GWAS summary statistics in BC over time. (**B**) Types of PGx studies in the TC over time. (**C**) Ancestries of participants in BC over time. (**D**) Ancestries of participants in the TC over time. (**E**) Pattern of single−/multi-trait in BC over time (‘Single Trait’ means a paper only uses one single trait for PRS analysis; ‘stPRS’ means a paper explores multiple traits one at a time; and ‘mtPRS’ means a paper construct PRS using multiple traits jointly).

Our initial search identified 834 papers from Medline and 2487 papers from EMBASE between 2013 and 2022. After pre-screening and combining the two databases, 127 papers were left for full-text screening. Ultimately, 90 papers were included in our systematic review (Figure S2, Table S1). Despite a steady increase in the number of PRS application articles between 2013 and 2022 (Figure 1), the PRS analysis in PGx GWAS still faces multiple challenges, which have been extensively discussed in the published overview papers [12–15] and briefly summarized in the previous section. In this paper, we focus on the three aforementioned key challenges in greater details.

### Challenge 1: Lack of knowledge about whether to choose PGx, disease or both GWAS summary statistics in the base cohort for PRS construction

The use of PRSs in PGx requires a BC and a TC. In the BC, summary statistics are generated to obtain risk variant effect sizes, standard errors and p-values to inform PRS calculations; in the TC, the PRS is developed and tested. To date, many researchers leverage GWAS derived from large cohorts of related disease and/or complex trait (i.e. non-disease) phenotypes in the development of polygenic models to predict drug outcome. For example, Figure 1A and B indicate that 74 out of 90 identified articles (~82%) published by 2022 use BC GWAS from disease studies. These include disease phenotypes related to the drug efficacy [8, 9, 26–68], complex trait phenotypes related to the drug efficacy [69–81], disease phenotypes related to the adverse drug reaction [82–91], and complex trait phenotypes related to the adverse drug reaction [92–96]. However, our previous research showed that such disease PRS approach cannot recover the full heritability of drug response unless an extremely stringent assumption that every causal variant has an interaction effect proportionate to its main effect is true [19]. More details are provided in the following section. Therefore, 16 out of 90 papers construct the PRSs for drug response prediction using PGx GWAS summary statistics directly instead of using disease GWAS summary statistics since the PGx variants are directly associated with drug response, which may provide larger power (especially in predicting drug response through the predictive effect of a PRS) [11, 97–111]. However, using PGx GWAS as the BC for PRS modeling presents unique challenges due to its typically small sample size, and the difficulty in identifying two cohorts with uniformly treated patients. These factors can lead to increased PRS modeling uncertainty and result in low PRS prediction power. To date, it remains unclear whether it is optimal to use disease GWAS summary statistics that emphasize disease variants with only prognostic effects, PGx GWAS summary statistics that emphasize PGx variants with both prognostic and predictive effects, or a combination of both in the BC for PRS construction and analysis in PGx GWAS.

### Challenge 2: Eurocentric or trans-ethnic bias in cross-population PRS prediction

Recent studies have found that PRSs exhibit reduced cross-population prediction accuracy, particularly in non-European populations [20, 21, 22]. Building PRSs with GWAS data from the same non-European population may yield limited prediction accuracy due to the typically small sample size of non-European GWAS compared with European GWAS. This applies to PRS applications in both disease GWAS and PGx GWAS. Conversely, constructing PRSs using European GWAS with larger sample size may offer limited improvement in prediction accuracy due to Eurocentric or trans-ethnic bias. This bias arises from differences in allele frequencies, LD patterns and effect sizes across populations [20, 21, 22]. With the increasing efforts to diversify genomic study samples, non-European genomic resources have been expanded, leading to a rapid growth in PGx studies leveraging multiple populations (Figure 1C and D), from 1 study in 2016 to 22 in 2021. For instance, Cearns *et al*. constructed a PRS using meta summary statistics calculated from seven cohorts with European, American, Asian and African populations [55]. To mitigate the impact of trans-ethnic bias, there is a pressing need to develop PRS methods that can handle such trans-ethnic data. Recent publications have introduced new methods for trans-ethnic PRS analysis in disease genetics [112, 113], but it is unclear whether these methods can be directly applied to PGx studies. To date, to our knowledge, no PGx PRS methods have been developed to address trans-ethnic drug response prediction under PGx settings.

### Challenge 3: Small sample size, low power and more complex PRS modeling in PGx GWAS

Subjects in the PGx GWAS are usually from RCTs, where the sample sizes are much smaller than those from large disease cohorts. Such small sample size typically results in low power for predicting drug responses with prognostic PRS components. There are many strategies to increase the power of PRS analysis in PGx GWAS. In this sense, integrating multiple traits during PGx PRS construction provides a natural way to increase power. To date, most PRSs are constructed using a single trait (i.e. using univariate disease or PGx GWAS summary statistics). However, most disease and PGx GWAS data are multivariate in nature with multiple correlated traits or drug responses. Intuitively, leveraging information from multiple correlated traits can potentially capture more genetic variance, thus boosting the power of PRS analysis. In fact, the number of published papers that explore multiple traits increased rapidly between 2019 and 2021 from 4 to 25 (Figure 1E). Specifically, 20 out of 25 papers analyze multiple PRSs separately. These PRSs are constructed from multiple diseases and/or complex traits that are potentially related to the drug response phenotype in the TC. For example, in Fanelli *et al*., the authors investigated the possible association of PRSs for bipolar disorder, major depressive disorder, neuroticism and schizophrenia with antidepressant non-response or non-remission in patients with major depressive disorder [28]. The remaining papers analyzed multiple PRSs jointly using either machine learning (ML) based regression or multivariate regression. For example, Taylor *et al*. used 10 PRSs for depression and genetically correlated traits as predictors in an elastic net model to predict response in tertiary care patients with resistant depression [81].

There are additional challenges to be addressed while constructing a multi-trait PRS in PGx GWAS compared with disease GWAS. First, a PGx multi-trait PRS requires more complex statistical modeling to handle prognostic and predictive effects simultaneously. In literature, very few papers explore this strategy. For example, Lanfear *et al*. [11] presented a successful PRS application example in PGx GWAS study, which built a PRS using 44 SNPs with predictive (or treatment-by-SNP interaction) effects only. Second, the genetic architecture is more complicated when considering different effect correlation relationships (e.g. magnitudes and directions) between multiple traits in the BC and the phenotype in the TC. It remains a question whether the current multi-trait PRS (mtPRS) approaches built for disease multi-trait PRS analysis are still robust when applied to PGx GWAS or not.

### Overview of the PRS methods applied in PGx GWAS

A large variety of methods are available for PRS construction and analysis. They differ from each other in terms of which variants and weights are used for constructing PRS and conducting PRS association analysis and/or prediction. Existing PRS methods generally fall into one of four categories: (i) clumping and thresholding (C + T) approaches, which shrink effect sizes of non-significant SNPs to zero according to their p-values, and account for LD by clumping variants at a given LD; (ii) methods that select variants jointly using penalized regression in the framework of ML, where the number of selected causal variants is controlled by the penalty parameter, and the LD matrix is intrinsic to the algorithm [114–118]; (iii) methods that account for LD through a linear mixed effects model, and estimate effect sizes as best linear unbiased predictions (BLUP) [119, 120, 121] and (iv) Bayesian approaches that explicitly model causal effects and LD to infer the posterior distribution of causal effect sizes, where the shrinkage is controlled by the prior distributions, and the LD matrix is integral to the algorithm [19, 112, 122–126]. Compared with simple C + T method, genome-wide model fitting with penalized or Bayesian regression generally better accounts for LD and has more efficient bias-variance trade-off, thus achieving better performance. In this paper, we summarized 25 PRS methods (including two novel methods/strategies we propose to tackle the challenges) using a three-level hierarchical structure (Table 1). On the first level, we categorized the methods based on the type of BC (i.e. using disease GWAS only, using PGx GWAS only or leveraging both disease and PGx GWAS summary statistics). On the second level, the methods are further categorized based on trait and ancestry information (i.e. single-trait single-ancestry; single-trait trans-ethnic; and multi-trait single-ancestry approaches). We do not consider the multi-trait trans-ethnic approaches since no such methods are currently available. On the third level, methods are divided into the four categories we mentioned before: C + T, ML, BLUP and Bayesian regression. More details about the 23 existing methods including their software sources are summarized in Table S2.

**Table 1:** Summary of 23 existing methods and two newly proposed analysis strategies and methods for PRS analysis, which are organized in a three-level hierarchical structure. On the first level, methods are categorized into using disease GWAS only, using PGx GWAS only, and leveraging both disease and PGx GWAS summary statistics. On the second level, methods are further divided into single-trait single-ancestry, single-trait trans-ethnic, and multi-trait single-ancestry approaches. On the third level, methods are assigned into four categories: C + T, ML, BLUP and Bayesian regression. The new strategies and methods [i.e. PRS-PGx-Bayes (Disease + PGx) and PRS-PGx-Bayesx] are highlighted in bold, which are proposed to address the challenges in the current PRS PGx methods. The number in the ‘Applications (# of papers)’ represents the total number of papers out of the 90 papers that use the method in that row. ‘0’ in the same column indicates this method is not from the 90 papers, but from other method (review) papers

GWAS	Scenario	Category	Method	Description	Required Data	Tuning Parameters	Applications (# of papers)
Disease GWAS	Single-trait single-ancestry	C + T	C + T	LD based clumping and p-value thresholding	Summary statistics	P-value threshold	60
		ML	Lassosum	Lasso regression based	Summary statistics	Penalty ($\mathrm{\lambda}$), s	1
			MegaPRS	A super learning algorithm choosing the best prediction model from Lasso, Ridge, and BOLT-LMM	Summary statistics	Parameters that maximize prediction for each model	0
		BLUP	SBLUP	BLUP in LMM	Summary statistics	NA	0
		Bayesian	LDpred	Bayesian shrinkage using point-normal prior	Summary statistics	Fraction of causal markers, Heritability	4
			LDpred2	Bayesian shrinkage method extended from LDpred, by enabling sparsity	Summary statistics	Fraction of causal markers, Heritability, Sparsity	1
			PRS-CS	Bayesian shrinkage method using global–local scale mixtures of normals prior	Summary statistics	Global shrinkage parameter ($\mathrm{\phi}$)	3
			DBSLMM	Bayesian shrinkage	Summary statistics	NA	0
			SBayesR	Bayesian shrinkage	Summary statistics	NA	0
	Single-trait trans-ethnic	C + T	CT-Meta	C + T based on meta-GWAS	Summary statistics	P-value threshold	22
		ML	TL-Multi	Lasso-regression-based method extended from Lassosum. Conducting transfer learning to learn information from EUR to correct the bias for non-EUR	Summary statistics	Penalty ($\mathrm{\lambda}$), s	0
			Multi-ethnic PRS	Combine training data from European samples and training data from the target population	Summary statistics	Mixing weights ${\mathrm{\alpha}}_1$ and ${\mathrm{\alpha}}_2$	0
		BLUP	XP-BLUP	Two-component LMM	Individual data	NA	0
		Bayesian	PRS-CSx	Bayesian shrinkage method extended from PRS-CS, by leveraging trans-ethnic populations	Summary statistics	Global shrinkage parameter ($\mathrm{\phi}$)	0
	Multi-trait single-ancestry	C + T	CT-Meta	C + T based on meta-GWAS	Summary statistics	P-value threshold	0
		ML	mtPRS-ML	Combine multiple PRS constructed by C + T from different traits with elastic-net regression	Individual data	P-value threshold, Penalty ($\mathrm{\lambda}$), $\mathrm{\alpha}$	2
			mtPRS-MultiReg	Combine multiple PRS constructed by C + T from different traits with multivariate regression	Individual data	NA	2
			CTPR	A cross-trait penalty function with the Lasso and the minimax concave penalty assuming the secondary trait is beneficial for predicting the primary trait	Individual data	Lasso penalty (${\mathrm{\lambda}}_1$), Minimax concave penalty (${\mathrm{\lambda}}_2$)	0
		BLUP	wMT-SBLUP	Extended from SBLUP for multiple traits, where weighted indices are calculated from SBLUP estimates	Summary statistics	NA	0
		Bayesian	–	–	–	–	–
PGx GWAS	Single-trait single-ancestry	C + T	PRS-PGx-CT	C + T on PGx GWAS summary statistics	Summary statistics	P-value threshold	0
		ML	PRS-PGx-L, GL, SGL	Penalized regression using Lasso, Group Lasso, and Sparse Group Lasso	Individual data	Penalty ($\mathrm{\lambda}$), s	0
		BLUP	–	–	–	–	–
		Bayesian	PRS-PGx-Bayes	Bayesian shrinkage method using two-dimensional global–local scale mixtures of normals prior based on PGx GWAS summary statistics	Summary statistics	Global shrinkage parameter ($\mathrm{\phi}$), v	0
	Single-trait trans-ethnic	Bayesian	**PRS-PGx-Bayesx**	Bayesian shrinkage method extended from PRS-PGx-Bayes, by leveraging trans-ethnic populations	Summary statistics	Global shrinkage parameter ($\mathrm{\phi}$), v	–
	Multi-trait single-ancestry	–	–	–	–	–	–
Disease and PGx GWAS	Single-trait single-ancestry	Bayesian	**PRS-PGx-Bayes** ** (Disease + PGx)**	Bayesian shrinkage method extended from PRS-PGx-Bayes, by leveraging joint information from both disease and PGx GWAS summary statistics	Summary statistics	Global shrinkage parameter ($\mathrm{\phi}$), v	–

**Figure 2:**
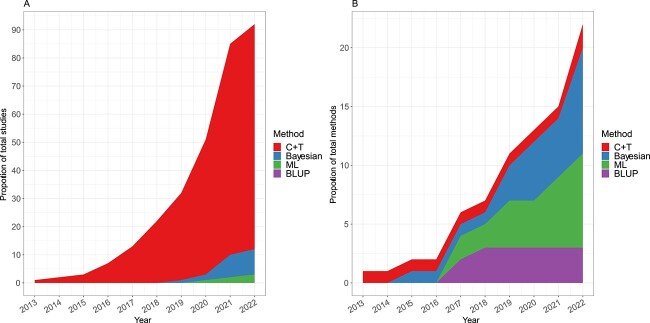
Number of PRS methods included in the review over time. (**A**) Number of papers stratified by four types of PRS methods (C + T, ML, BLUP and Bayesian) applied to PGx GWAS over time. The total number of papers across different methodologies is larger than 90 since some papers explored multiple methods. (**B**) Number of PRS methods developed over time, stratified by four types of PRS methods.

Figure 2A presents an overview of the methods used in the 90 articles identified in this study. As of March 11, 2022, 91.1% (82/90) of the papers employed the C + T method for PRS construction, which is simple and user-friendly but may discard informative SNPs, potentially limiting the prediction power. A small proportion of papers (8/90 = 8.9%) utilized Bayesian methods, including PRS-CS, LDpred and LDpred2. Only two papers (2/90 = 2.2%) used ML based approaches, and no study investigated BLUP methods. Despite the prevalence of the C + T method in these studies, the use of more complex Bayesian and ML algorithms has been increasing since 2014, accounting for 39.1% and 34.8% of studies from 2022, respectively (Figure 2B). This trend reflects a growing interest in employing more advanced PRS modeling methods to improve performance in both disease and PGx GWAS.

## TACKLING CHALLENGE 1: LEVERAGING BOTH PGX AND DISEASE GWAS SUMMARY STATISTICS IN THE BASE COHORT FOR PRS CONSTRUCTION IN PGX PRS APPLICATIONS

### Using disease GWAS in the base cohort may lead to poor drug response prediction performance

**Figure 3:**
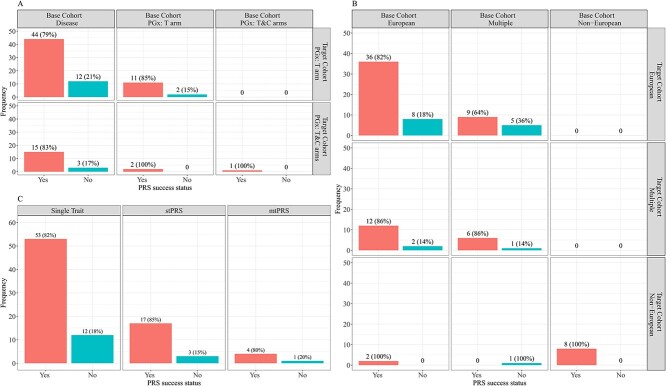
PRS application success rates. (**A**) Success rates under different combinations of study types between BC and TC. (**B**) Success rates under different combinations of ancestries between BC and TC and (**C**) Success rates stratified by different types of traits. If a paper has ‘PRS success status = Yes’, then that paper has identified a statistically significant association between the PRS and the endpoint in the TC.

PGx PRS applications can be categorized into three types in terms of the BC GWAS type: disease GWAS, the PGx GWAS with treatment arm only, and the full PGx GWAS with two arms (treatment and control); and two types in terms of the TC study type: the PGx study with treatment arm only, and the PGx study with two arms. Using disease GWAS in the BC and then applying it to PGx studies represents the largest percentage (74/90 = 82%) of 90 identified papers, possibly due to the wide publicly available resources of disease GWAS summary statistics, and the difficulty in finding PGx GWAS data with the same or similar drug response endpoints. However, as we noted before, such disease PRS approach can barely recover the full heritability of drug response, which is consistent with what we report in Figure 3A. Specifically, the use of disease GWAS in the BC results in the largest failure rate (defined as the proportion of the papers with no significant association found between the PRS and the drug responses): 20% (15/74); when the BC GWAS are from PGx studies, the failure rate decreases to 6% (2/16). This is not surprising since most papers using disease GWAS-based PRSs in PGx applications rely on the assumption that if a variant is a significant signal for some specific disease, then that variant is also causal for the response of the drug targeting that disease. However, the underlying genetic correlation between complex trait/disease risk and drug response needs to be carefully investigated and may vary case by case. Moreover, more studies now tend to focus on PGx studies with two arms and perform the interaction test of differential treatment effect between the high and low genetic risk subgroups when stratified by the PRS. Many such successful findings have been reported [11, 110].

### Disease PRS is not able to recover the full heritability of drug response in theory

We have previously proved that, in theory, it is difficult for a disease PRS to recover the full heritability of a drug response in PGx GWAS [19]. Here, we briefly summarize the theoretical derivation and the main conclusions. Consider a high-dimensional regression model where the drug response, after adjusting for the covariates, is determined by three components: treatment, genotype and genotype-by-treatment interaction. Furthermore, to capture the correlation between prognostic and predictive effects, we assume the two effects follow a multivariate-normal distribution. Under these assumptions, we derive the squared correlation coefficient between a disease PRS (${\mathrm{S}}_{\mathrm{dis}}={\sum}_{\mathrm{j}=1}^{\mathrm{m}}{\mathrm{G}}_{\mathrm{j}}{\mathrm{\beta}}_{\mathrm{j}}$) and the drug response ($\mathrm{Y}$) for the treated subjects, denoted by $\mathrm{co}{\mathrm{r}}^2\left({\mathrm{S}}_{\mathrm{dis}},\mathrm{Y}\right)$. We proved that by the Cauchy-Schwarz inequality, $\mathrm{co}{\mathrm{r}}^2\left({\mathrm{S}}_{\mathrm{dis}},\mathrm{Y}\right)\le{\mathrm{h}}^2$, where ${\mathrm{h}}^2$ denotes the underlying heritability of the drug response studied. It directly showed that a disease PRS cannot recover the full genetic variability of a drug response unless the equality holds. We further proved that the equality holds if and only if the interaction effect is proportional to the main effect for every causal variant, which is a very stringent assumption under PGx settings. In [19], the authors used a specific example with a real PGx GWAS data from the IMPROVE-IT RCT [127] to calculate $\mathrm{co}{\mathrm{r}}^2\left({\mathrm{S}}_{\mathrm{dis}},\mathrm{Y}\right)={\mathrm{h}}^2\left(1-0.54\right)$, which demonstrated that any PRSs developed from disease GWAS explained at most 46% variability of the LDL-C drug response. This real data example highlights the importance of switching to using PGx GWAS/variants (like what [19] does) or the combination of PGx and disease GWAS/variants as the BC in the PGx PRS modeling and analysis.

### Leveraging both PGx and disease GWAS in the base cohort for improving drug response prediction

It is worth noting that there are certain limitations when calculating PRSs using PGx GWAS summary statistics only. First, it can be challenging to find two independent PGx GWAS data with same or similar traits. Second, the sample size in PGx studies is usually small, which may lead to limited prediction power. In fact, Zhai *et al*. [19] showed in their simulation studies that PGx PRS methods did not necessarily outperform disease PRS approaches in the control arm. In other words, if we only focus on the control arm of a PGx study, a disease PRS may still be useful compared to a PGx PRS due to its large sample size, which provides greater power in PRS prediction in terms of the prognostic effect component. However, in real scenario we may be more interested in the treatment arm or both treatment and control arms. Therefore, a compromise and a direct alterative solution is to use both PGx GWAS data and disease GWAS summary statistics for PRS construction in the BC if both are available. In this section, we aim to compare different combinations between two PRS construction strategies of using summary statistics (either disease or PGx GWAS only or both disease and PGx GWAS) and four PRS methods (C + T, Lassosum, PRS-CS and PRS-PGx-Bayes). Eight approaches will be compared, which are listed in Table 2. In this table, we mainly focus on how to incorporate strategies of leveraging summary statistics into PRS methods. And the description details of the methods themselves can be found in Table S2.

**Table 2:** Summary of eight combinations between two PRS construction strategies of using summary statistics (either disease or PGx GWAS only or both disease and PGx GWAS) and four PRS methods (C + T, Lassosum, PRS-CS and PRS-PGx-Bayes). Detailed descriptions of methods can be found in Table S2

		Strategies of using different summary statistics	
		Using either disease or PGx GWAS only	Using both disease and PGx GWAS
PRS Methods	C + T	C + T (Disease): Train the C + T model using disease GWAS summary statistics only from BC. After the p-value shrinkage, the algorithm outputs prognostic effect sizes of SNPs ${\mathrm{\beta}}_{\mathrm{G}}$. The PRS is calculated as $\mathrm{PRS}_{\mathrm{G}}=\mathrm{G}{\mathrm{\beta}}_{\mathrm{G}}$. And the drug response is predicted as $\mathrm{E}\left[\mathrm{Y}\right]={\mathrm{\beta}}_0+{\mathrm{\beta}}_1\mathrm{T}+{\mathrm{\beta}}_2\mathrm{PRS}_{\mathrm{G}}+{\mathrm{\beta}}_3\mathrm{T}\times \mathrm{PRS}_{\mathrm{G}}$.	C + T (Disease + PGx): Train the C + T model using disease GWAS summary statistics and PGx GWAS summary statistics with genotype-by-treatment interaction information, respectively, from BC. After the p-value shrinkage, the algorithm outputs prognostic effect sizes of SNPs ${\mathrm{\beta}}_{\mathrm{G}}$ and predictive effect sizes of SNPs ${\mathrm{\beta}}_{\mathrm{GT}}$ from disease and PGx GWAS, respectively. Two PRSs are calculated as $\mathrm{PRS}_{\mathrm{G}}=\mathrm{G}{\mathrm{\beta}}_{\mathrm{G}}$ and $\mathrm{PR}{\mathrm{S}}_{\mathrm{GT}}=\mathrm{G}{\mathrm{\beta}}_{\mathrm{GT}}$. And the drug response is predicted as $\mathrm{E}\left[\mathrm{Y}\right]={\mathrm{\beta}}_0+{\mathrm{\beta}}_1\mathrm{T}+{\mathrm{\beta}}_2\mathrm{PRS}_{\mathrm{G}}+{\mathrm{\beta}}_3\mathrm{T}\times \mathrm{PRS}_{\mathrm{G}\mathrm{T}}$.
	Lassosum	Lassosum (Disease): Similar to the above C + T method, train the Lassosum penalized regression model using disease GWAS summary statistics only from the BC.	Lassosum (Disease + PGx): Similar to the above C + T method, train the Lassosum penalized regression model using disease GWAS summary statistics and PGx GWAS summary statistics with genotype-by-treatment information, respectively, from the BC.
	PRS-CS	PRS-CS (Disease): Similar to the above C + T method, train the PRS-CS Bayesian regression model using disease GWAS summary statistics only from the BC.	PRS-CS (Disease + PGx): Similar to the above C + T method, train the PRS-CS Bayesian regression model under disease GWAS summary statistics and PGx GWAS summary statistics with genotype-by-treatment information, respectively, from the BC.
	PRS-PGx-Bayes	PRS-PGx-Bayes (PGx): Train the PRS-PGx-Bayes model using PGx GWAS summary statistics with genotype and genotype-by-treatment information jointly from BC. After the global–local shrinkage, the algorithm outputs prognostic effect sizes of SNPs ${\mathrm{\beta}}_{\mathrm{G}}$ and predictive effect sizes of SNPs ${\mathrm{\beta}}_{\mathrm{GT}}$. Two PRSs are calculated as $\mathrm{PR}{\mathrm{S}}_{\mathrm{G}}=\mathrm{G}{\mathrm{\beta}}_{\mathrm{G}}$ and $\mathrm{PR}{\mathrm{S}}_{\mathrm{GT}}=\mathrm{G}{\mathrm{\beta}}_{\mathrm{GT}}$. And the drug response is predicted as $\mathrm{E}\left[\mathrm{Y}\right]={\mathrm{\beta}}_0+{\mathrm{\beta}}_1\mathrm{T}+{\mathrm{\beta}}_2\mathrm{PRS}_{\mathrm{G}}+{\mathrm{\beta}}_3\mathrm{T}\times \mathrm{PRS}_{\mathrm{G}\mathrm{T}}$.	PRS-PGx-Bayes (Disease + PGx): Replace the genotype information in PGx GWAS summary statistics with disease GWAS summary statistics and keep the genotype-by-treatment information in PGx GWAS summary statistics unchanged. Then train the PRS-PGx-Bayes model under this composite summary statistics from the BC.

### Simulation studies for evaluating the new strategy by leveraging both PGx and disease GWAS in the base cohort for PRS construction in PGx GWAS

We performed extensive simulation studies to compare the performance of methods that use disease GWAS summary statistics only, PGx GWAS summary statistics only, or both. We simulated genotype data using R package sim1000G v1.40 with different sample sizes. To simulate prognostic ${\mathrm{\beta}}_{\mathrm{G}}$ and predictive ${\mathrm{\beta}}_{\mathrm{GT}}$ effect sizes, we used the same spike-and-slab distribution as described in Zhai *et al*. [19]. The two effects were either correlated (i.e. a causal variant had both non-zero prognostic and non-zero predictive effects) or fully separated (i.e. a causal variant had either non-zero prognostic or non-zero predictive effect). The prognostic effect is either on the same scale as, dominated by, or dominating the predictive effect. Details of the data generation process are provided in Supplementary Method A.

**Figure 4:**
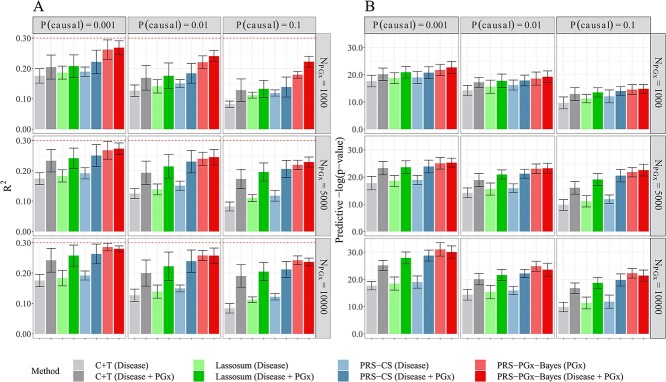
Methods comparison results (with internal cross-validation) among methods that use either disease GWAS summary statistics only, PGx GWAS summary statistics only, or both. The prognostic and predictive effects were correlated, and in the same scale. Heritability was fixed at 0.3. The numbers of the causal variants for P(causal) = 0.001, 0.01 and 0.1 were 23, 226 and 2263, respectively. The PGx GWAS summary statistics in the BC were calculated with 1000, 5000 and 10 000 subjects; the disease GWAS summary statistics in the BC were calculated with 50 000 subjects. The tuning parameters were selected via cross-validation in the TC. The performance was assessed in terms of (**A**) prediction accuracy R^2^, and (**B**) predictive p-value for the two-sided PRS-by-treatment interaction test. Data were presented as mean values +/− standard deviations (error bars) with 10 000 replications, where results were calculated from the TC.

Figure 4 shows the scenario where prognostic and predictive effects are correlated, and the two effect sizes are on the same scale. Results were assessed via internal 5-fold cross-validation in the TC. Specifically, the whole TC was randomly split into five folds. The tuning parameters were determined using four folds, and the PRS was constructed and recorded in the remaining fold. In Figure 4, we initially employed disease PRS methods (C + T, Lassosum, PRS-CS) using disease GWAS summary statistics only as the base. Subsequently, we incorporated additional PGx data to check the potential improvement of disease PRS methods. Similarly, we utilized PGx PRS method (PRS-PGx-Bayes) using PGx GWAS summary statistics only as the base, and then added additional disease genetics data to assess the improvement. All disease PRS and PGx PRS methods showed substantial improvement in the drug response prediction and patient stratification after leveraging additional GWAS information compared to their counterpart traditional approach using either disease or PGx GWAS alone. For example, PRS-PGx-Bayes (Disease + PGx) generally outperformed PRS-PGx-Bayes (PGx), especially under the higher polygenicity scenario when the sample size of PGx GWAS data was small. In addition, when the sample size of PGx GWAS data was large enough (for example, *n* = 10 000), PRS-PGx-Bayes (PGx) was superior to PRS-PGx-Bayes (Disease + PGx) as shown in both Figure 4 with internal cross-validation and Figure S3 with external validation (i.e. the optimal tuning parameter was selected using an independently simulated validation PGx dataset with sample size 1000 as shown in Supplementary Method A.5). One possible explanation is that it is due to the difference in the prognostic effect estimate between disease and PGx GWAS. The same pattern was also observed when the proportion of causal SNPs increased from 0.001 to 0.1, although all methods generally had lower performance.

Sensitivity analyses results are provided in Figures S3–S5. Specifically, in Figure S3, we repeated our comparisons using external validation, where the tuning parameters were selected with an independent validation dataset. The same patterns held compared to the case where internal validation was used. Figure S4 shows the scenario where the prognostic and predictive effect sizes were on different scales. When the prognostic effect dominated the predictive effect, it is not surprising that incorporating disease genetics data into the PGx PRS approach had a much larger improvement than incorporating PGx data into disease PRS methods. When the predictive effect dominated the prognostic effect, PRS-PGx-Bayes (Disease + PGx) consistently achieved the highest prediction accuracy. Figure S5 checks the situation when the prognostic and predictive effects were fully separated. In that setting, the disease PRS methods using disease GWAS summary statistics alone had the lowest ${\mathrm{R}}^2$ since they could hardly capture any variability explained by the interaction. Thus, incorporating PGx GWAS information greatly improved the performance of disease PRS methods.

## TACKLING CHALLENGE 2: REDUCING EUROCENTRIC BIAS USING A NEW TRANS-ETHNIC PGX PRS METHOD (PRS-PGX-BAYESX)

### Overview of the cross-population PRS applications in PGx GWAS

PGx PRS applications could be classified into three categories based on the BC ancestry: European, non-European (e.g. Asian, African) and Multiple (which is the mixture of, in most cases, European and TC non-European population); and three categories based on the TC ancestry: European, non-European and Multiple. No published articles were found to build PRSs with non-European ancestry alone in the BC unless the TC population is also non-European (the percentage is 8/90 = 9% in Figure 3B). On the other hand, applying a PRS built from European ancestry to the European population in the TC (which is referred to as ‘European + European’) remains the largest proportion (44/90 = 49%) of 90 selected studies, which is not surprising. In addition, Multiple + European doesn’t result in a higher success rate (defined as the proportion of the papers with significant associations found between the PRSs and the drug responses) compared to European + European (9/14 = 64% versus 36/44 = 82%). One possible explanation is that adding non-European subjects may add more noise relative to the amount of additional information. When the TC population is non-European, a trans-ethnic PRS analysis method is needed since a traditional PRS built on European population has limited transferability across ancestry groups [20, 21, 22], and a PRS built on non-European GWAS might yield reduced prediction accuracy due to its small sample size. However, among the 90 articles identified, only one paper followed this trans-ethnic PRS strategy, and failed to find any significant association between PRSs and the drug response. Due to the limited exploration of trans-ethnic analysis in PGx applications, it may imply great potential opportunities for applying cross-population analysis in future PGx studies.

### A new trans-ethnic PGx PRS method (PRS-PGx-Bayesx) for cross-population PRS analysis in PGx GWAS

A few PRS methods have been published for trans-ethnic analysis in disease genetics, which can be adapted to the PGx setting. A brief description of these methods is listed below, using two populations, EUR and EAS, as an example.

1. CT-Meta: C + T based on Meta-GWAS summary statistics, which is calculated by aggregating two disease GWAS summary statistics from EUR and EAS together.
2. Multi-ethnic PRS [117]: $\mathrm{PR}{\mathrm{S}}_0=\mathrm{\pi} \times \mathrm{PR}{\mathrm{S}}_{\mathrm{EUR}}+\left(1-\mathrm{\pi} \right)\times \mathrm{PR}{\mathrm{S}}_{\mathrm{EAS}}$, which uses a grid to search the optimal $\mathrm{\pi}$ using cross-validation.
3. PRS-CSx [112]: a method extended from PRS-CS method for trans-ethnic analysis via a shared continuous shrinkage prior across different populations.

However, to our knowledge, no method has been adapted to trans-ethnic PGx PRS analysis yet. In this study, we propose a novel method (PRS-PGx-Bayesx) which is an extension of the PRS-PGx-Bayes method [19] for the cross-population PRS construction and analysis.

Consider $\mathrm{K}$ high-dimensional Bayesian regression models of $\mathrm{n}$ patients and $\mathrm{m}$ SNPs from $\mathrm{K}$ studies (or populations in the trans-ethnic GWAS case):


$$ {\mathrm{Y}}_{\mathrm{k}}={\mathrm{G}}_{\mathrm{k}}{\mathrm{\beta}}_{\mathrm{k}}+\left({\mathrm{G}}_{\mathrm{k}}\times{\mathrm{T}}_{\mathrm{k}}\right){\mathrm{\alpha}}_{\mathrm{k}}+{\mathrm{\varepsilon}}_{\mathrm{k}},{\mathrm{\varepsilon}}_{\mathrm{k}}\sim \mathrm{N}\left(0,{\mathrm{\sigma}}_{\mathrm{k}}^2\right),\mathrm{k}=1,\cdots, \mathrm{K}, $$

where ${\mathrm{Y}}_{\mathrm{k}},{\mathrm{T}}_{\mathrm{k}},{\mathrm{G}}_{\mathrm{k}}$ denote the drug response, the binary treatment assignment, and the $\mathrm{n}\times \mathrm{m}$ genotype matrix in study $\mathrm{k}$, respectively. ${\mathrm{\beta}}_{\mathrm{jk}}$ and ${\mathrm{\alpha}}_{\mathrm{jk}}$ are the prognostic and predictive effects of SNP $\mathrm{j}$ in study $\mathrm{k}$, respectively.

The regression coefficient ${\mathrm{b}}_{\mathrm{jk}}=\left({\mathrm{\beta}}_{\mathrm{jk}},{\mathrm{\alpha}}_{\mathrm{jk}}\right)$ is assumed to be random in the PRS-PGx-Bayesx method, following a prior distribution:


$$\left(\begin{array}{c}{\mathrm{\beta}}_{\mathrm{j}\mathrm{k}}\\{}{\mathrm{\alpha}}_{\mathrm{j}\mathrm{k}}\end{array}\right)\sim \mathrm{MVN}\left(0,\frac{\mathrm{\sigma}_{\mathrm{k}}^2}{{\mathrm{N}}_{\mathrm{k}}}\mathrm{\phi} {\mathrm{M}}_{\mathrm{j}}\right),\mathrm{where}\ {\mathrm{M}}_{\mathrm{j}}=\left[\begin{array}{cc}{\mathrm{\psi}}_{\mathrm{j}}& {\mathrm{\rho}}_{\mathrm{j}}\sqrt{\mathrm{\psi}_{\mathrm{j}}{\mathrm{\xi}}_{\mathrm{j}}}\\{}{\mathrm{\rho}}_{\mathrm{j}}\sqrt{\mathrm{\psi}_{\mathrm{j}}{\mathrm{\xi}}_{\mathrm{j}}}& {\mathrm{\xi}}_{\mathrm{j}}\end{array}\right],$$

$\mathrm{\phi}$ controls the overall degree of shrinkage, while ${\mathrm{\psi}}_{\mathrm{j}}$ and ${\mathrm{\xi}}_{\mathrm{j}}$ control the marker-specific degree of shrinkage. Both $\mathrm{\phi}$ and ${\mathrm{\psi}}_{\mathrm{j}},{\mathrm{\xi}}_{\mathrm{j}}$ are shared across all $\mathrm{K}$ studies. Further, assume the residual variance ${\mathrm{\sigma}}_{\mathrm{k}}^2$ follows a non-informative scale-invariant Jeffreys prior, that is, $\mathrm{p}\left({\mathrm{\sigma}}_{\mathrm{k}}^2\right)\propto 1/{\mathrm{\sigma}}_{\mathrm{k}}^2$. As suggested by Zhai *et al*. [19], we propose to use the hierarchical half-t prior [128] on the variance–covariance matrix ${\mathrm{M}}_{\mathrm{j}}$:


\begin{align*}& {\mathrm{M}}_{\mathrm{j}}\sim{\mathrm{W}}^{-1}\left({\mathrm{B}}_{\mathrm{j}},2\mathrm{v}+1\right),\mathrm{where}\ {\mathrm{B}}_{\mathrm{j}}=4\mathrm{v}\left(\begin{array}{cc}{\mathrm{\delta}}_{\mathrm{j}}& 0\\{}0& {\mathrm{\lambda}}_{\mathrm{j}}\end{array}\right),\\ & {\mathrm{\delta}}_{\mathrm{j}}\sim \mathrm{G}\left({\mathrm{b}}_1,1\right),{\mathrm{\lambda}}_{\mathrm{j}}\sim \mathrm{G}\left({\mathrm{b}}_2,1\right), \end{align*}

${\mathrm{W}}^{-1}\left({\mathrm{B}}_{\mathrm{j}},2\mathrm{v}+1\right)$ denotes the inverse Wishart distribution with scale matrix ${\mathrm{B}}_{\mathrm{j}}$ and $\mathrm{v}$ degrees of freedom, and G denotes the Gamma distribution.

Given the above prior information, we can derive posterior distributions:


\begin{align*} &{\mathrm{b}}_{\mathrm{k}}\!\mid\! \mathrm{Y}\sim \mathrm{MVN}\left({\mathrm{\mu}}_{\mathrm{k}},{\Sigma}_{\mathrm{k}}\right), \mathrm{where}\ {\mathrm{\mu}}_{\mathrm{k}}\!=\!\frac{{\mathrm{N}}_{\mathrm{k}}}{\sigma_{\mathrm{k}}^2}{\Sigma}_{\mathrm{k}}{\hat{\mathrm{b}}}_{\mathrm{k}},\mathrm{and}\!\ {\Sigma}_{\mathrm{k}}\!=\!\frac{\sigma_{\mathrm{k}}^2}{{\mathrm{N}}_{\mathrm{k}}}{\left({\mathrm{D}}_{\mathrm{k}}\!+\!{\Omega}^{-1}\right)}^{-1}\\ &{\mathrm{D}}_{\mathrm{k}}={\mathrm{X}}_{\mathrm{k}}^{\prime }{\mathrm{X}}_{\mathrm{k}}/{\mathrm{N}}_{\mathrm{k}}=\left[\begin{array}{cc}\mathrm{cor}\left({\mathrm{G}}_{\mathrm{k}},{\mathrm{G}}_{\mathrm{k}}\right)& \mathrm{cor}\left({\mathrm{G}}_{\mathrm{k}},{\mathrm{G}}_{\mathrm{k}}\times \mathrm{T}\right)\\{}\mathrm{cor}\left({\mathrm{G}}_{\mathrm{k}}\times \mathrm{T},{\mathrm{G}}_{\mathrm{k}}\right)& \mathrm{cor}\left({\mathrm{G}}_{\mathrm{k}}\times \mathrm{T},{\mathrm{G}}_{\mathrm{k}}\times \mathrm{T}\right)\end{array}\right],\\ & \mathrm{where}\ {\mathrm{X}}_{\mathrm{k}}=\left[{\mathrm{G}}_{\mathrm{k}}\ {\mathrm{G}}_{\mathrm{k}}\times{\mathrm{T}}_{\mathrm{k}}\right]\Omega =\left[\begin{array}{cc}\Psi & \mathrm{P}\!\!\!\! \\{}\mathrm{P} & \Xi\!\!\!\! \end{array}\right],\Psi =\operatorname{diag}\left({\mathrm{\psi}}_{\mathrm{j}}\right),\Xi =\operatorname{diag}\left({\mathrm{\xi}}_{\mathrm{j}}\right),\\ & \mathrm{P}=\operatorname{diag}\left({\mathrm{\rho}}_{\mathrm{j}}\sqrt{\psi_{\mathrm{j}}{\mathrm{\xi}}_{\mathrm{j}}}\right){\mathrm{\sigma}}_{\mathrm{k}}^2\mid \mathrm{Y}\sim \mathrm{iG}\\ & \left(\mathrm{M}+\frac{{\mathrm{N}}_{\mathrm{k}}}{2},\frac{{\mathrm{N}}_{\mathrm{k}}}{2}\left[1-2{\hat{\mathrm{b}}}_{\mathrm{k}}^{\prime }{\mathrm{b}}_{\mathrm{k}}+{\mathrm{b}}_{\mathrm{k}}^{\prime}\left({\mathrm{D}}_{\mathrm{k}}+{\Omega}^{-1}\right){\mathrm{b}}_{\mathrm{k}}\right]\right),\\ & \mathrm{where}\ \mathrm{iG}=\mathrm{inverse}\ \mathrm{G}\mathrm{amma}{\mathrm{M}}_{\mathrm{j}}\mid \left({\mathrm{b}}_{\mathrm{j}\mathrm{k}},\mathrm{k}=1,\cdots, \mathrm{K}\right)\\ & \sim{\mathrm{W}}^{-1}\left({\mathrm{B}}_{\mathrm{j}}+{\mathrm{A}}_{\mathrm{j}},2\mathrm{v}+\mathrm{K}+1\right),\mathrm{where}\ {\mathrm{A}}_{\mathrm{j}}=\sum_{\mathrm{k}=1}^{\mathrm{K}}\frac{{\mathrm{N}}_{\mathrm{k}}}{\sigma_{\mathrm{k}}^2}\left[\begin{array}{cc}{\mathrm{\beta}}_{\mathrm{j}\mathrm{k}}^2& {\mathrm{\beta}}_{\mathrm{j}\mathrm{k}}{\mathrm{\alpha}}_{\mathrm{j}\mathrm{k}}\\{}{\mathrm{\beta}}_{\mathrm{j}\mathrm{k}}{\mathrm{\alpha}}_{\mathrm{j}\mathrm{k}}& {\mathrm{\alpha}}_{\mathrm{j}\mathrm{k}}^2 \end{array}\!\right]\\ & {\mathrm{\delta}}_{\mathrm{j}}\mid{\mathrm{M}}_{\mathrm{j}}\sim \mathrm{G}\left(\mathrm{v}+{\mathrm{b}}_1+\frac{1}{2},\mathrm{\phi} +\frac{2\mathrm{v}}{\psi_{\mathrm{j}}\left(1-{\mathrm{\rho}}_{\mathrm{j}}^2\right)}\right),\\&{\mathrm{\lambda}}_{\mathrm{j}}\mid{\mathrm{M}}_{\mathrm{j}}\sim \mathrm{G}\left(\mathrm{v}+{\mathrm{b}}_2+\frac{1}{2},\mathrm{\phi} +\frac{2\mathrm{v}}{\xi_{\mathrm{j}}\left(1-{\mathrm{\rho}}_{\mathrm{j}}^{2.}\right)}\right) \end{align*}

It is worth noting that, as derived in Zhai *et al*. [19], LD reference panel of population k is required to calculate ${\mathrm{D}}_{\mathrm{k}}$, for each $\mathrm{k}=1,\cdots, \mathrm{K}$. Detailed theoretical derivation is provided in Supplementary Method B, and the detailed algorithm of PRS-PGx-Bayesx method is summarized in Algorithm 1. We ran extensive simulations to compare the proposed PRS-PGx-Bayesx method with existing trans-ethnic PRS methods mentioned above.

**Table:** 

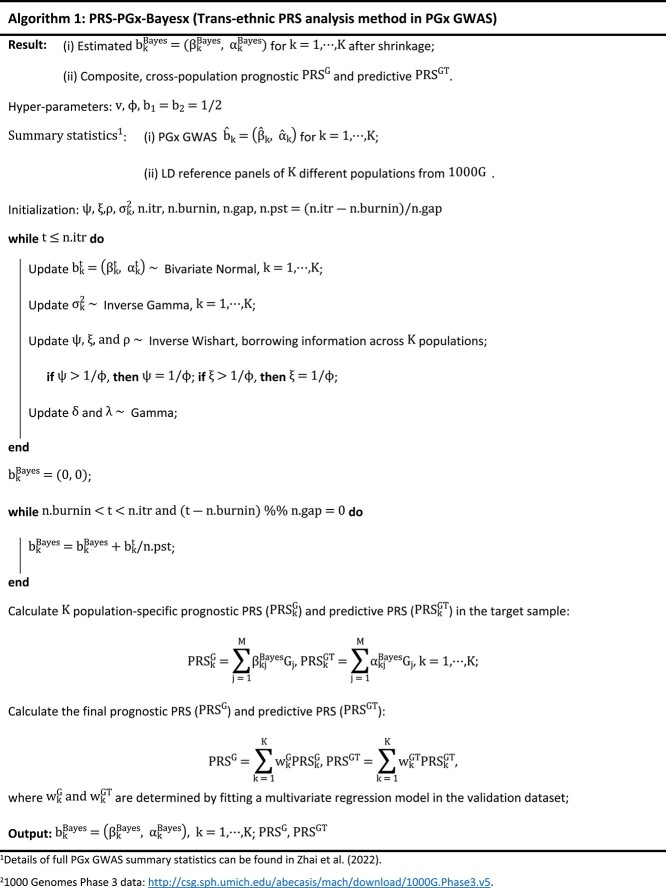

### Simulation studies for evaluating the proposed trans-ethnic PGx PRS method (PRS-PGx-Bayesx)

In this section, we performed extensive simulation studies to compare performances of multiple trans-ethnic methods with single-ethnic methods when the TC population is either European (EUR) or East Asian (EAS). Simulation studies were performed with simulated genotype data using sim1000G software for both EUR and EAS. We used a vector of parameters $\mathrm{v}=\left({\mathrm{\mu}}_1,{\mathrm{\mu}}_2,{\mathrm{\gamma}}_1,{\mathrm{\gamma}}_2,{\mathrm{\beta}}_1,{\mathrm{\beta}}_2,{\mathrm{\alpha}}_1,{\mathrm{\alpha}}_2\right)$ to denote the underlying true prognostic ($\mathrm{\mu}, \mathrm{\beta}$)/predictive ($\mathrm{\gamma}, \mathrm{\alpha}$) effect size in base ($\mathrm{\mu}, \mathrm{\gamma}$)/target ($\mathrm{\beta}, \mathrm{\alpha}$) cohort of European (with index 1)/non-European (with index 2) population. Following the simulation setup by Ruan *et al*. [112], we further assumed $\mathrm{v}$ follows a multivariate-normal distribution with a well-defined variance–covariance matrix $\Sigma \left({\mathrm{\rho}}_{\mathrm{E}},{\mathrm{\rho}}_{\mathrm{P}},{\mathrm{\rho}}_{\mathrm{C}}\right)$, where ${\mathrm{\rho}}_{\mathrm{E}}$ measures the correlation between prognostic and predictive effects, ${\mathrm{\rho}}_{\mathrm{P}}$ measures the effect correlation between European and non-European populations, ${\mathrm{\rho}}_{\mathrm{C}}$ measures the effect correlation between BC and TC. Details of the data generation process are provided in Supplementary Method C.

**Figure 5:**
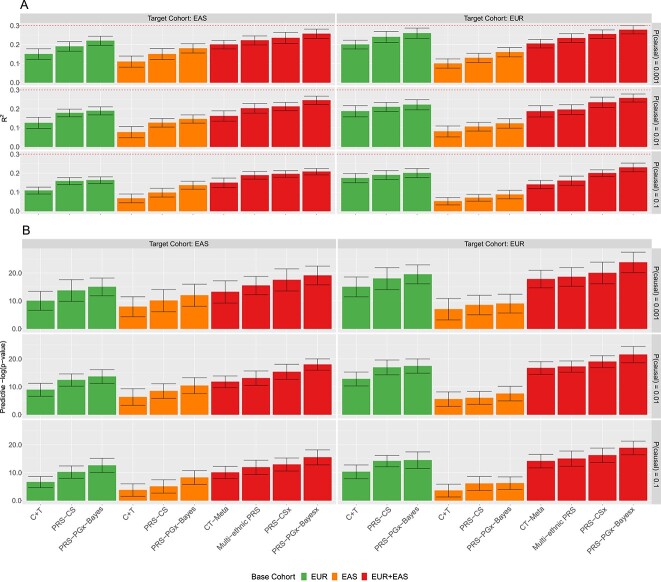
Methods comparisons between single-ethnic methods (using EUR or EAS alone) and trans-ethnic methods (using EUR and EAS jointly) when the effect correlation between EUR and EAS populations ${\mathrm{\rho}}_{\mathrm{P}}=0.5$. The prognostic and predictive effects were correlated, and in the same scale. Heritability was fixed at 0.3. The numbers of the causal variants for P(causal) = 0.001, 0.01 and 0.1 were 12, 119 and 1188, respectively, for EUR population; and were 11, 106 and 1065, respectively, for EAS population. In the BC, the disease GWAS summary statistics of EUR and EAS populations were calculated based on 50 000 and 10 000 subjects, respectively; the PGx GWAS summary statistics of EUR and EAS populations were calculated based on 5000 and 1000 subjects, respectively. Sample sizes of EUR and EAS populations in the TC were 5000 and 1000, respectively. The tuning parameters were selected via cross-validation in the TC. The performance was assessed in terms of (**A**) prediction accuracy R^2^, and (**B**) predictive p-value for the two-sided PRS-by-treatment interaction test. Data were presented as mean values +/− standard deviations (error bars) with 10 000 replications, where results were calculated from the TC.

Figure 5 shows that under our simulation setup (i.e. EUR-EAS effect correlation ${\mathrm{\rho}}_{\mathrm{P}}=0.5$), constructing PRSs using EUR alone outperforms using EAS alone even when the TC is EAS. And using EAS in the BC to predict EUR TC results in the smallest ${\mathrm{R}}^2$ and the largest interaction p-value. When the TC is EUR, using EUR + EAS in the BC has limited improvements compared to using EUR alone due to the small sample size of EAS and the moderate correlation between EUR and EAS. On the other hand, when the TC is EAS, trans-ethnic analysis of EUR + EAS is superior to using EAS alone clearly by incorporating the information of EUR population. Lastly, PRS-PGx-Bayesx method is superior to all the other methods for drug response prediction across different proportions of causal variants.

For sensitivity analysis, we considered scenarios when the EUR-EAS effect correlation is low (${\mathrm{\rho}}_{\mathrm{P}}=0.1$; Figure S6) and when the EUR-EAS effect correlation is high (${\mathrm{\rho}}_{\mathrm{P}}=0.9$; Figure S7). When ${\mathrm{\rho}}_{\mathrm{P}}=0.1$, using EUR for the prediction of EAS and using EAS for the prediction of EUR both retained the lowest prediction accuracy. Furthermore, trans-ethnic predictions are not necessarily superior to single-ethnic ones, since integrating EAS in the BC to predict EUR in the TC may include noise rather than signals, and vice versa. When ${\mathrm{\rho}}_{\mathrm{P}}=0.9$, all approaches have better performance. Both Figures S6 and S7 indicate that our proposed novel PRS-PGx-Bayesx still outperforms other methods.

## TACKLING CHALLENGE 3: USING MULTI-TRAIT PRS METHODS TO INCREASE POWER FOR PRS ANALYSIS IN PGX GWAS

We categorized the 90 selected papers into three categories: those using a single trait, those exploring multiple traits one at a time, and those building PRSs by aggregating multiple traits together. Figure 3C indicates that the three approaches had similar success rates of 82% (53/65), 85% (17/20) and 80% (4/5), respectively. This is possibly due to the very small number of the multi-trait PRS papers. With the success of leveraging information from multiple related traits for signal detection in GWAS [129] and WES (SNP-set analysis) [130], it is also appealing to use multiple traits to increase the power of PRS analysis for drug response prediction and patient stratification in PGx GWAS. Our review results in Figure 3C show that some PGx GWAS are starting to use multi-trait PRS methods. However, it remains unclear which methods are robust under different genetic architectures in PGx studies.

There are a variety of multi-trait PRS analysis methods in literature. For example, the regression-based methods [42, 71, 80, 81] fit a linear or a penalized regression model with individual PRSs from multiple traits as predictors; the meta-GWAS-based methods [131, 132] construct the PRSs using meta-GWAS summary statistics by aggregating individual GWAS summary statistics from multiple traits; the BLUP-based method (wMT-SBLUP) [121] combines the single-trait predictors with BLUP properties in a weighted index calculated from genome-wide SNP heritability, genetic correlation between traits, and expected squared correlations between the phenotype and BLUP predictors; the PCA-based method (mtPRS-PCA) [24] combines PRSs from multiple traits with weights obtained from performing PCA on the genetic correlation matrix, etc. In addition, a more robust method mtPRS-O [24] combines several complimentary multi-trait PRS methods via Cauchy Combination Test. With a variety of multi-trait PRS analysis methods developed, there is an urgent need to systematically evaluate their robustness to different genetic architectures. Zhai *et al*. [24] provided a comprehensive simulation framework and ran extensive simulations to systematically compare most of the multi-trait PRS methods under various genetic architectures covering different effect directions, signal sparseness and cross-trait correlation structures. Table S3 briefly summarizes the main features of the existing multi-trait PRS methods in terms of their pros, cons and performances in simulation studies from [24] and our additional simulation analyses (the detailed results are not shown). In terms of the PRS association test, mtPRS-O is the most robust method that achieves optimally larger power compared with other multi-trait methods; however, in terms of the drug response prediction, no single method uniformly outperforms the others across all scenarios. In summary, integrating multiple genetically correlated traits from disease GWAS does increase the power for PRS based drug response prediction and patient stratification in PGx GWAS.

## DISCUSSION

In this study, we systematically review 90 PRS application papers in PGx GWAS for drug response prediction and patient stratification. We summarize 23 PRS methodologies from these 90 PRS application papers and three other PRS method review papers. From this review, we show that although both PRS application and PRS method development have progressed rapidly in PGx fields, the PRS analysis in PGx GWAS still faces multiple challenges from the PRS analysis method standpoint. In this paper, we mainly dive into three key challenges: (i) the lack of the knowledge on choosing PGx, disease or both GWAS summary statistics in the BC for PRS construction; (ii) the Eurocentric or trans-ethnic bias in the cross-population PRS prediction; (iii) the small sample size, low power and more complex PRS modeling in PGx GWAS. We further propose two new PRS analysis strategies and methods to tackle these challenges.

For the first challenge, under PGx settings, we compare traditional disease GWAS-based methods (C + T, Lassosum, PRS-CS) and PGx GWAS-based method (PRS-PGx-Bayes), with our proposed novel strategy of leveraging both disease and PGx GWAS summary statistics to construct PRS, PRS-PGx-Bayes (Disease + PGx). It is an extension of PRS-PGx-Bayes by replacing the prognostic effect size estimates from PGx GWAS summary statistics with the effect size estimates from disease GWAS summary statistics, which is simple and easy to be implemented. In the simulation studies, we find that the combination of disease GWAS based PRS analysis methods and additional information from PGx GWAS improves the drug response prediction accuracy. Moreover, PRS-PGx-Bayes (Disease + PGx) generally outperforms the other methods especially when the sample size of PGx GWAS in the BC is not large enough. Intuitively, using PGx GWAS summary statistics allows us to model the prognostic effect (i.e. genotype main effect) and predictive effect (i.e. genotype-by-treatment interaction effect) simultaneously, while including disease GWAS summary statistics from disease genetics studies generally provides a much larger sample size than PGx studies, which improves the modeling of the prognostic effect component of a PGx PRS. For the second challenge about the Eurocentric or trans-ethnic bias, although there are already some PRS methods in literature from the disease genetics field, their performance in PGx GWAS setting is unknown. To reduce the Eurocentric or trans-ethnic prediction bias, we propose a novel method, PRS-PGx-Bayesx, which is an extension of PRS-PGx-Bayes for trans-ethnic analysis by updating the global–local shrinkage parameters with cross-population information. Specifically, we assume that the variance–covariance matrix of prognostic and predictive effects of SNP j (${\mathrm{M}}_{\mathrm{j}}$) is shared across all K studies/populations, and the posterior distribution of ${\mathrm{M}}_{\mathrm{j}}$ is derived conditional on all K studies. Our simulation studies indicate that PRS-PGx-Bayesx is superior to other methods regardless of the correlation between European and non-European populations. For the third challenge, we focus on reviewing the methods which integrate multiple traits during PGx PRS construction and provide a natural way of increasing power for PRS analysis in PGx GWAS. In addition to the review of the applications of mtPRS methods in PGx GWAS, we further refer to our previous research work [24] and provide a comprehensive summary of the performance of the existing multi-trait performance in PGx GWAS.

Our study has some limitations. First, in this review, the study screening was conducted by two independent reviewers (Z.S. and S.J.) to minimize selection bias. However, there is currently no risk of bias assessment tool for PRS related reviews [133]. Therefore, some evidence may still be missing due to publication bias (e.g. negative results are less likely to be reported) [14, 134, 135]. Second, our efforts of tackling the first challenge demonstrate the fact that leveraging both disease and PGx GWAS summary statistics may further improve the power of PGx PRS analysis. However, it would be more challenging to obtain both disease GWAS and PGx GWAS with similar (or genetic correlated) phenotypes. In addition, it is difficult to evaluate the genetic correlation relationship between a disease phenotype and a relevant drug response phenotype (i.e. by calculating their genetic correlation). Our proposed solution by replacing ${\mathrm{\beta}}_{\mathrm{G}}$ of the PGx GWAS with ${\mathrm{\beta}}_{\mathrm{G}}$ from the disease GWAS is simple and intuitive. However, there is a gap between the prognostic effect sizes from disease GWAS and those from PGx GWAS. Therefore, our simple strategy may not increase the prediction accuracy, especially when the sample size of the PGx GWAS is large enough (we have demonstrated this point in the simulation results summarized in Figure 4). In theory, more complicated models can be constructed for further increasing the PRS prediction performance. However, they may face additional barriers in clinical interpretation and implementation. In addition, the PRS-PGx-Bayesx method we develop for tackling the second challenge is a Bayesian based method, which requires a relatively longer computational time (as shown in Figure S8). Therefore, it is recommended to perform PRS-PGx-Bayesx method by LD blocks [19, 124, 136] under the high performance computing or parallel computing environment. Besides, PRS-PGx-Bayesx is an extension from PRS-PGx-Bayes [19] for cross-population analysis, and it shares the same challenges of the PRS-PGx-Bayes method. For example, PRS-PGx-Bayesx uses one of the most popular continuous shrinkage priors, the global–local scale mixtures of normals (i.e. the Horseshoe prior), for effect size shrinkage. There are other candidate priors (e.g. the spike-and-slab prior and the Normal-Gamma shrinkage prior), and the currently existing Bayesian methods do not have a systematic way to determine the optimal prior. Third, we focus our study on the three main challenges in current PRS analyses under PGx settings. Other challenges mentioned before (e.g. the lack of guidance in clinical interpretation of PGx polygenic models) are beyond the scope of this paper, but have been discussed in other PRS PGx review papers [12–15]. Fourth, phenotypic characterization also presents a unique challenge within pharmacogenomic research as many drug outcomes are difficult to measure quantitatively [14]. Discrepant phenotyping may lead to different polygenic models being constructed depending on the definition of the drug outcome since they will result in different effective sample sizes and power for PRS analyses and predictions. Finally, with the rapid development of PRS applications and methodologies as we show in this paper, more efforts are needed to accelerate the PGx PRS application with clinical utilities. Further research should now aim at comparing the drug response prediction accuracy with and without the use of PRSs to demonstrate the benefit of PRSs in PGx applications.

As this field of research continues to grow, we believe that there are many promising applications for the use of PRSs in the context of PGx and precision medicine to further improve treatment outcomes. Our efforts of reviewing the current progress of PRS applications and methods in PGx GWAS, identifying the current main challenges and proposing new analysis strategies and methods to further overcome them in PGx PRS applications help move a step forward in the field and may accelerate the translation of PRSs to clinical practice.

**Key Points**

- The application of PRSs in PGx GWAS has begun to show great potentials for improving patient stratification and drug response prediction. However, applying PRSs to PGx GWAS faces multiple challenges including (i) the lack of knowledge about whether PGx, disease or both GWAS/variants should be used in the BC; (ii) the Eurocentric or trans-ethnic bias; (iii) small sample sizes in PGx GWAS with low power and (iv) the more complex PRS modeling while handling both prognostic and predictive effects simultaneously, etc.
- We conduct a systematic review of current progress in both PRS applications and PRS method developments in PGx GWAS to gain insights about the general trends, challenges and possible solutions.
- We further propose (i) a novel PRS application strategy by leveraging both PGx and disease GWAS summary statistics in the BC for PRS construction in PGx PRS applications and (ii) a new Bayesian method (PRS-PGx-Bayesx) to reduce Eurocentric or across-population PRS prediction bias. Our extensive simulations demonstrate their advantages over existing PRS methods applied in PGx GWAS.
- Our systematic review and methodology research work in this paper not only highlights current gaps and key considerations while applying PRS methods to PGx GWAS, but also provides possible solutions for better PGx PRS applications and future research.

## Supplementary Material

PGxPRS_Review_ms_Supp_Final_bbad470

Table_S1_S2_bbad470

## DATA AND MATERIALS AVAILABILITY

1000 Genomes Phase3 v5 data: http://csg.sph.umich.edu/abecasis/mach/download/1000G.Phase3.v5.html. sim1000G R package v1.40: https://cran.r-project.org/web/packages/sim1000G/index.html. HapMap3 data: https://www.ncbi.nlm.nih.gov/variation/news/NCBI_retiring_HapMap/. LD blocks in human population: https://bitbucket.org/nygcresearch/ldetect-data/src/master. Lassosum: https://github.com/tshmak/lassosum. PRS-CS: https://github.com/getian107/PRScs. PRS-CSx: https://github.com/getian107/PRScsx. mtPRS-PCA and mtPRS-O: https://github.com/zhaiso1/mtPRS. PRS-PGx-Bayes R package v0.3.0: https://cran.r-project.org/web/packages/PRSPGx/index.html. R code we used to summarize our review results and the PRS-PGx-Bayesx R function are freely available at https://github.com/zhaiso1/PGxPRSOverview.

## Author Biographies

**Song Zhai** is a Senior Scientist, Biostatistics and Research Decision Sciences, Merck Sharp & Dohme LLC, a subsidiary of Merck & Co., Inc., Rahway, NJ, USA. He holds a PhD in Statistics, and his current research focuses on biomarker discovery in pharmacogenomics and disease studies.

**Devan V. Mehrotra** is Vice President, Biostatistics and Research Decision Sciences at Merck Sharp & Dohme LLC, a subsidiary of Merck & Co., Inc., Rahway, NJ, USA. His current research focus is on statistical methods for stratified medicine.

**Judong Shen** is a Senior Director, Biostatistics and Research Decision Sciences, Merck Sharp & Dohme LLC, a subsidiary of Merck & Co., Inc., Rahway, NJ, USA. His research focuses on developing statistical methods for data analysis in pharmacogenomics, disease genetics, drug target science and translational biomarker studies.
