# Supplementary material for: Applying polygenic risk score methods to pharmacogenomics GWAS: challenges and opportunities
Source: Brief Bioinform. 2023 Dec 27;25(1):bbad470. doi: 10.1093/bib/bbad470 (PMC10782924; doi:10.1093/bib/bbad470)
Supplement: PGxPRS_Review_ms_Supp_Final_bbad470 [file pgxprs_review_ms_supp_final_bbad470.pdf]

Supplementary Materials for

Applying polygenic risk score methods to pharmacogenomics GWAS: challenges and opportunities

Song Zhai[1], Devan V. Mehrotra[2], Judong Shen[1*]

[1]Biostatistics and Research Decision Sciences, Merck & Co., Inc., Rahway, NJ 07065, USA

[2]Biostatistics and Research Decision Sciences, Merck & Co., Inc., North Wales, PA 19454, USA

* To whom correspondence should be addressed

Correspondence: judong.shen@merck.com

This file includes:

Supplementary Method

Figure S1 to S8

Table S3

Supplementary Reference

Other Supplementary Materials for this manuscript include the following:

Table S1

Table S2

Supplementary Methods

A. Data generation process for simulating different GWAS summary statistics

A.1 Simulate genotype data

We performed simulation studies using simulated genotype data from R package sim1000G v1.40 with different sample sizes. The sim1000G software simulated variants in genomic regions among unrelated individuals with initial population from 1000 Genomes (1000G) Phase 3 European samples (n = 503). We used the HapMap3 CEU sample (n = 234) as an external LD reference panel. 22,630 SNPs on chromosome 19 were left after matching between the 1000G and the HapMap3 datasets.

A.2 Simulate prognostic and predictive effect sizes

The SNP prognostic and predictive effect sizes were simulated jointly with the following distribution:

$$\begin{pmatrix} \beta_j^{(k)} \\ \alpha_j^{(k)} \end{pmatrix} \sim \begin{cases} \mathrm{MVN}(0, \Sigma_k) & \text{with probability } \pi_k, \\ 0 & \text{with probability } 1 - \pi_k. \end{cases}$$

where $\pi_k \sim \mathrm{Beta}(p, 1-p)$, p denoted the proportion of causal SNPs, j was the index of j-th SNP, and k was the index of k-th LD block [1]. Furthermore,

$$\Sigma_k = \begin{bmatrix} \psi & \rho_k\sqrt{\psi\xi} \\ \rho_k\sqrt{\psi\xi} & \xi \end{bmatrix}, \text{where } \rho_k \sim \mathrm{Uniform}(0,1).$$

$\psi/\xi = 1$ implied that the prognostic and predictive effects were in the same scale, while $\psi/\xi \neq 1$ implied that one effect was dominant to another effect. It was worth noting that the above simulation indicates each causal SNP carried some degree of prognostic effect and some degree of predictive effect. To generate completely separated prognostic and predictive SNPs, we randomly chose half of the causal variants and only kept their prognostic effects (i.e., artificially shrank $\alpha_j = 0$); for the rest half of the causal variants, only predictive effects were kept (i.e., artificially shrank $\beta_j = 0$).

A.3 Simulate disease and PGx GWAS summary statistics in the base cohort

To simulate disease GWAS data in the base cohort, the phenotype was generated as:

$$Y_{n\times 1} = G_{n\times m}\beta_{m\times 1} + \epsilon_{n\times 1},$$

where n = 50,000 and m = 22,630. Then we calculated disease GWAS summary statistics with the simulated individual-level data. To simulate PGx GWAS data in the base cohort, the phenotype was generated as:

$$Y_{n\times 1} = \beta_T T_{n\times 1} + G_{n\times m}\beta_{m\times 1} + (G \times T)_{n\times m}\alpha_{m\times 1} + \epsilon_{n\times 1}, \quad (1)$$

where n = 1,000, 5,000, or 10,000 and m = 22,630. Then we calculated PGx GWAS summary statistics with the simulated individual-level data.

A.4 Simulate PGx GWAS data in the target cohort

The data generation process described in A.3 (Equation 1) was repeated to generate 5,000 individual-level PGx GWAS data with two arms (1-to-1 ratio) from randomized clinical trial (RCT).

A.5 Simulate PGx GWAS data for validation

The data generation process described in A.3 (Equation 1) was repeated to generate 1,000 individual-level PGx GWAS data with two arms (1-to-1 ratio) from randomized clinical trial (RCT). This dataset was used as the validation data for selecting the optimal tuning parameters.

B. Derivation of posterior distributions in the proposed PRS-PGx-Bayesx method

Consider K high-dimensional Bayesian regression models of $\mathrm{N_k}$ patients and M SNPs from K studies (or populations):

$$Y_k = G_k\beta_k + (G_k \times T_k)\alpha_k + \epsilon_k, \epsilon_k \sim N(0, \sigma_k^2), p(\sigma_k^2) \propto \frac{1}{\sigma_k^2}, k = 1, \cdots, K.$$

$$\begin{pmatrix}\beta_{jk}\\ \alpha_{jk}\end{pmatrix} \sim MVN\left(0, \frac{\sigma_k^2}{N_k}M_j\right), \text{where } M_j = \begin{bmatrix}\psi_j & \rho_j\sqrt{\psi_j\xi_j}\\ \rho_j\sqrt{\psi_j\xi_j} & \xi_j\end{bmatrix}.$$

$$M_j \sim W^{-1}(B_j, 2v+1), \text{where } B_j = 4v\begin{pmatrix}\delta_j & 0\\ 0 & \lambda_j\end{pmatrix}, \delta_j \sim G(b_1, \phi), \lambda_j \sim G(b_2, \phi).$$

B.1 Posterior distributions of $\mathbf{b_k}$ and $\boldsymbol{\sigma_k^2}$

For each $k \in 1, \cdots, K$,

$$\begin{aligned}
p(b_k, \sigma_k^2 | Y_k) &\propto p(\sigma_k^2)p(b_k|\sigma_k^2)p(Y_k|\beta_k, \sigma_k^2)\\
&\propto (\sigma_k^2)^{-1}(\sigma_k^2)^{-M}e^{-\frac{N_k}{2\sigma_k^2}b_k'\Omega^{-1}b_k}(\sigma_k^2)^{-\frac{N_k}{2}}e^{-\frac{1}{2\sigma_k^2}(Y_k-X_kb_k)'(Y_k-X_kb_k)}\\
&\propto (\sigma_k^2)^{-\left(M+\frac{N_k}{2}+1\right)}e^{-\frac{N_k}{2\sigma_k^2}\left[b_k'\Omega^{-1}b_k+\frac{1}{N_k}(Y_k-X_kb_k)'(Y_k-X_kb_k)\right]}\\
&\propto (\sigma_k^2)^{-\left(M+\frac{N_k}{2}+1\right)}e^{-\frac{N_k}{2\sigma_k^2}\left[b_k'\Omega^{-1}b_k+\frac{1}{N_k}Y_k'Y_k-\frac{2}{N_k}Y_k'X_kb_k+\frac{1}{N_k}b_k'X_k'X_kb_k\right]},
\end{aligned}$$

where $b_k = (\beta_k, \alpha_k)$, $X_k = [G_k \quad G_k \times T_k]$, and $\Omega = \begin{bmatrix}\Psi & P\\ P & \Xi\end{bmatrix}, \Psi = \mathrm{diag}(\psi_j), \Xi = \mathrm{diag}(\xi_j), P = \mathrm{diag}\left(\rho_j\sqrt{\psi_j\xi_j}\right)$. Assume 1) Y_k is standardized so that $Y_k'Y_k/N_k = 1$; 2) the prognostic and predictive effect sizes from PGx GWAS summary statistics $\hat{b}_k = (\hat{\beta}_k, \hat{\alpha}_k) = X_k'Y_k/N_k$; 3) $D_k = X_k'X_k/N_k$, we have

$$p(b_k, \sigma_k^2|Y_k) \propto (\sigma_k^2)^{-\left(M+\frac{N_k}{2}+1\right)}e^{-\frac{N_k}{2\sigma_k^2}[b_k'(D_k+\Omega^{-1})b_k+1-2\hat{b}_k'b_k]}.$$

Now we can already see that

$$\sigma_k^2|Y \sim iG\left(M + \frac{N_k}{2}, \frac{N_k}{2}\left[1 - 2\hat{b}_k'b_k + b_k'(D_k + \Omega^{-1})b_k\right]\right), \text{where iG denotes the inverse Gamma distribution.}$$

Notice that

$$\begin{aligned}
&b_k'(D_k + \Omega^{-1})b_k - 2\hat{b}_k'b_k\\
&= \left(b_k - (D_k + \Omega^{-1})^{-1}\hat{b}_k\right)'(D_k + \Omega^{-1})\left(b_k - (D_k + \Omega^{-1})^{-1}\hat{b}_k\right) - \hat{b}_k'(D_k + \Omega^{-1})^{-1}\hat{b}_k,
\end{aligned}$$

therefore

$$b_k|Y \sim MVN(\mu_k, \Sigma_k), \text{where } \mu_k = \frac{N_k}{\sigma_k^2}\Sigma_k\hat{b}_k, \text{and } \Sigma_k = \frac{\sigma_k^2}{N_k}(D_k + \Omega^{-1})^{-1}.$$

B.2 Posterior distribution of M_j

The posterior distribution of M_j borrows the information across K studies:

$$\begin{aligned} p(M_j|b_{jk}, k = 1, \cdots, K) &\propto p(b_{jk}, k = 1, \cdots, K|M_j)p(M_j) \\ &\propto p(M_j)\prod_{k=1}^{K} p(b_{jk}|M_j) \\ &\propto |M_j|^{-\frac{2v+1+p+1}{2}} e^{-\frac{1}{2}tr(B_jM_j^{-1})} \prod_{k=1}^{K}\left\{|M_j|^{-\frac{1}{2}} e^{-\frac{N_k}{2\sigma_k^2}b_{jk}'M_j^{-1}b_{jk}}\right\} \\ &\propto |M_j|^{-\frac{2v+K+1+p+1}{2}} e^{-\frac{1}{2}\left[tr(B_jM_j^{-1})+\sum_{k=1}^{K}\frac{N_k}{\sigma_k^2}b_{jk}'M_j^{-1}b_{jk}\right]} \\ &\propto |M_j|^{-\frac{2v+K+1+p+1}{2}} e^{-\frac{1}{2}\left[tr(B_jM_j^{-1})+\sum_{k=1}^{K} tr\left(\frac{N_k}{\sigma_k^2}b_{jk}b_{jk}'M_j^{-1}\right)\right]} \\ &\propto |M_j|^{-\frac{2v+K+1+p+1}{2}} e^{-\frac{1}{2}\left[tr\left[\left(B_j+\sum_{k=1}^{K}\frac{N_k}{\sigma_k^2}b_{jk}b_{jk}'\right)M_j^{-1}\right]\right]}. \end{aligned}$$

Therefore

$$M_j|(b_{jk}, k = 1, \cdots, K) \sim W^{-1}(B_j + A_j, 2v + K + 1), \text{where } A_j = \sum_{k=1}^{K}\frac{N_k}{\sigma_k^2}b_{jk}b_{jk}' = \sum_{k=1}^{K}\frac{N_k}{\sigma_k^2}\begin{bmatrix}\beta_{jk}^2 & \beta_{jk}\alpha_{jk} \\ \beta_{jk}\alpha_{jk} & \alpha_{jk}^2\end{bmatrix}.$$

B.3 Posterior distributions of δ_j and λ_j

$$\begin{aligned} p(\delta_j, \lambda_j|M_j) &\propto p(M_j|\delta_j, \lambda_j)p(\delta_j, \lambda_j) \\ &\propto |B_j|^{\frac{2v+1}{2}} e^{-\frac{1}{2}tr(B_jM_j^{-1})}\delta_j^{b_1-1}e^{-\phi\delta_j}\lambda_j^{b_2-1}e^{-\phi\lambda_j} \end{aligned}$$

Therefore

$$\delta_j\Big|M_j \sim G\left(v + b_1 + \frac{1}{2}, \phi + \frac{2v}{\psi_j(1-\rho_j^2)}\right), \lambda_j\Big|M_j \sim G\left(v + b_2 + \frac{1}{2}, \phi + \frac{2v}{\xi_j(1-\rho_j^2)}\right).$$

C. Data generation process for trans-ethnic simulation studies

C.1 Simulate genotype data

Simulation studies were performed with simulated genotype data using sim1000G v1.40 software for both EUR and EAS. The 1000G Phase 3 EUR (n = 503) and EAS (n = 504) samples were served as inputs of initial populations for sim1000G. We used HapMap3 CEU (n = 234) and JPT+CHB (n = 340) as external LD reference panels for European and East Asian populations, respectively. 22,630 SNPs on chromosome 19 were left for analysis after matching between HapMap3 CEU and the 1000G EUR. Similarly, 20,290 SNPs on chromosome 19 were left after matching between HapMap3 JPT+CHB and the 1000G EAS.

C.2 Simulate prognostic and predictive effect sizes

We used a vector of parameters $(\mu_1, \mu_2, \gamma_1, \gamma_2)$ to denote the underlying true prognostic (μ)/predictive (γ) effect size of European (with index 1)/non-European (with index 2) population in the base cohort. Similarly, we used a vector of parameters $(\beta_1, \beta_2, \alpha_1, \alpha_2)$ to denote the underlying true prognostic (β)/predictive (α) effect size of European/non-European population in the target cohort. For each SNP j, we assumed $(\mu_{j1}, \mu_{j2}, \gamma_{j1}, \gamma_{j2}, \beta_{j1}, \beta_{j2}, \alpha_{j1}, \alpha_{j2})$ follows the multivariate-normal distribution:

$$\begin{pmatrix} \mu_{j1} \\ \mu_{j2} \\ \gamma_{j1} \\ \gamma_{j2} \\ \beta_{j1} \\ \beta_{j2} \\ \alpha_{j1} \\ \alpha_{j2} \end{pmatrix} | R_j = \begin{cases} MVN(0, \Sigma), & R_j = 1 \\ 0, & R_j = 0 \end{cases}, \quad R_j \sim \text{Bernoulli}(p).$$

Furthermore,

$$\Sigma = \frac{h^2}{mp}\Omega, \text{and } \Omega = \begin{bmatrix} \Omega_1 & \Omega_2 \\ \Omega_2 & \Omega_1 \end{bmatrix}, \text{where}$$

$$\Omega_1 = \begin{bmatrix} 1 & \rho_P & \rho_E & \rho_P\rho_E \\ \rho_P & 1 & \rho_P\rho_E & \rho_E \\ \rho_E & \rho_P\rho_E & 1 & \rho_P \\ \rho_P\rho_E & \rho_E & \rho_P & 1 \end{bmatrix}, \text{and } \Omega_2 = \begin{bmatrix} \rho_C & \rho_C\rho_P & \rho_C\rho_E & \rho_C\rho_E\rho_P \\ \rho_C\rho_P & \rho_C & \rho_C\rho_E\rho_P & \rho_C\rho_E \\ \rho_C\rho_E & \rho_C\rho_E\rho_P & \rho_C & \rho_C\rho_P \\ \rho_C\rho_E\rho_P & \rho_C\rho_E & \rho_C\rho_P & \rho_C \end{bmatrix}.$$

Here, ρ_E measures the correlation between prognostic and predictive effects, ρ_P measures the correlation between European and non-European populations, and ρ_C measures the correlation between base and target cohorts.

C.3 Simulate disease and PGx GWAS summary statistics in the base cohort

We repeated Supplementary Method A.3 for EUR and EAS populations, respectively. Specifically, in the base cohort, the disease GWAS summary statistics of EUR and EAS populations were calculated based on 50,000 and 10,000

subjects, respectively; the PGx GWAS summary statistics of EUR and EAS populations were calculated based on 5,000 and 1,000 subjects, respectively.

C.4 Simulate PGx GWAS data in the target cohort

We repeated Supplementary Method A.4 for EUR and EAS populations, respectively. Sample sizes of EUR and EAS populations in the target cohort were 5,000 and 1,000, respectively.

Supplementary Figures

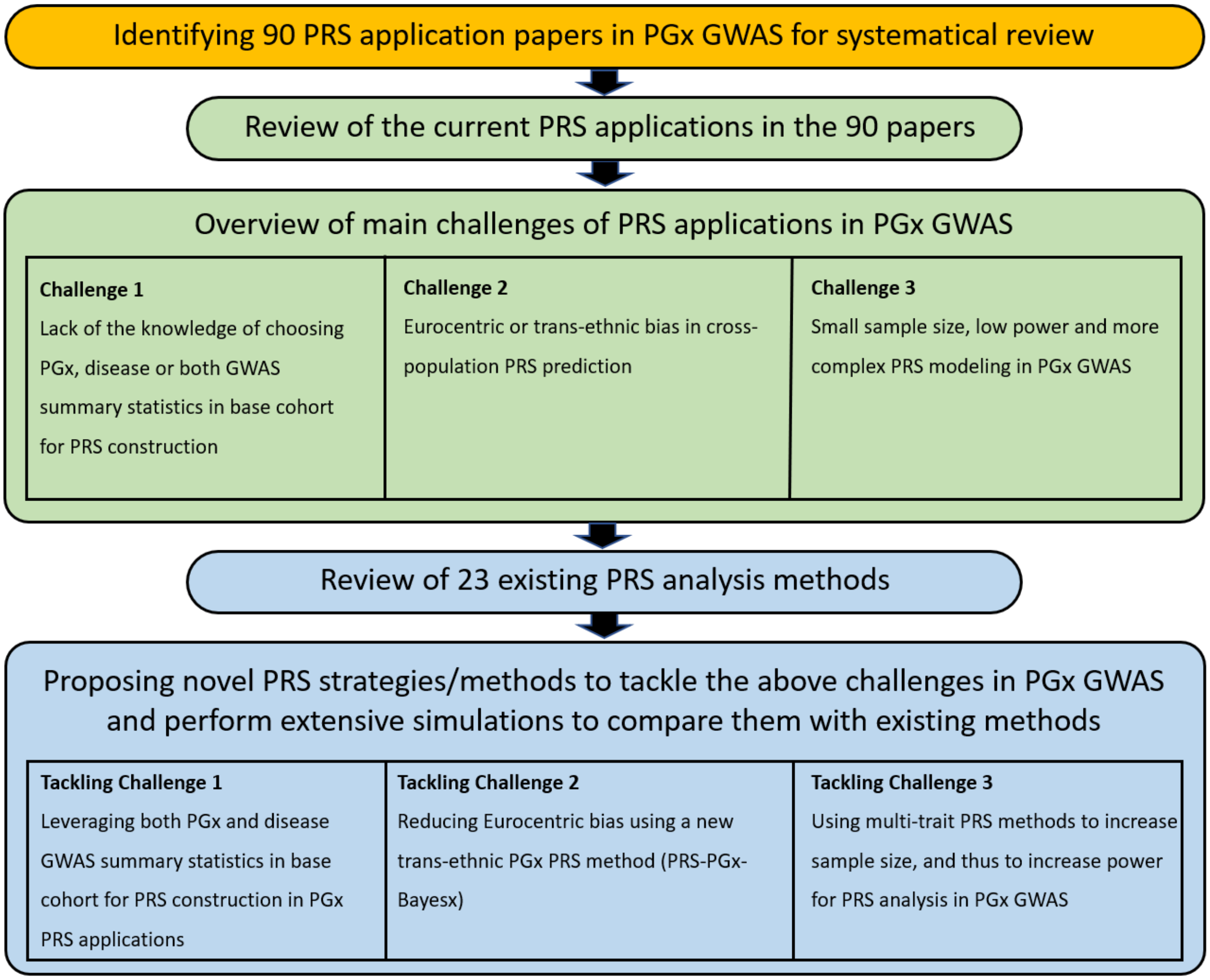

Figure S1: The overall workflow of the paper. The whole workflow includes five main steps: (1) Identifying 90 PRS application papers in PGx studies for systematical review; (2) Review of the current PRS applications in the 90 papers; (3) Overview of main challenges of PRS applications in PGx GWAS; (4) Review of 23 existing PRS analysis methods; (5) Proposing novel PRS strategies/methods to tackle challenges in PGx GWAS and perform extensive simulations to compare them with existing methods.

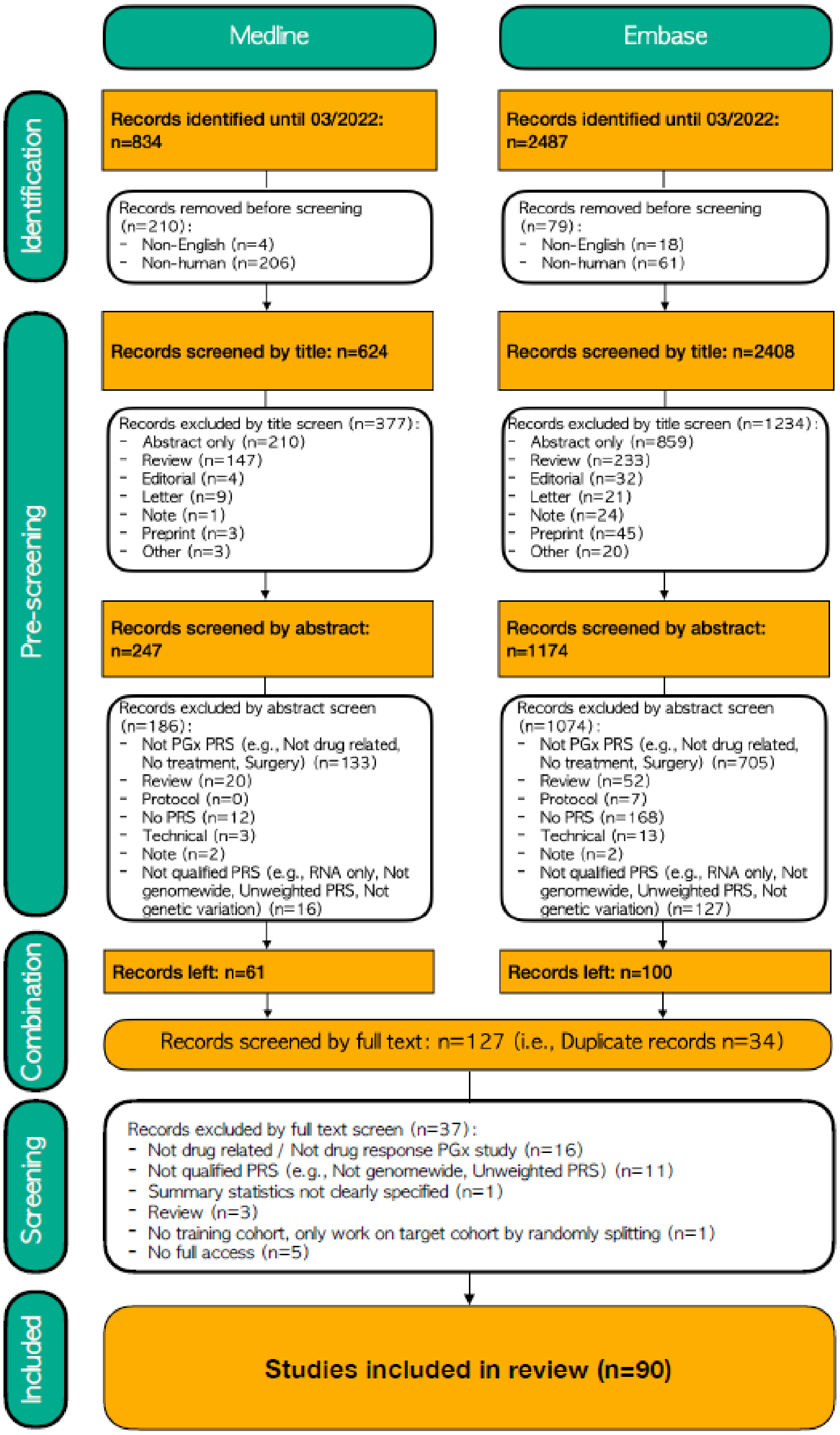

Figure S2: Workflow of identifying papers for review. The whole workflow includes four steps: (1) identification of records from Medline and Embase, respectively; (2) pre-screening by title and abstract; (3) combination of records together and removing duplicate records; and (4) screening based on full text.

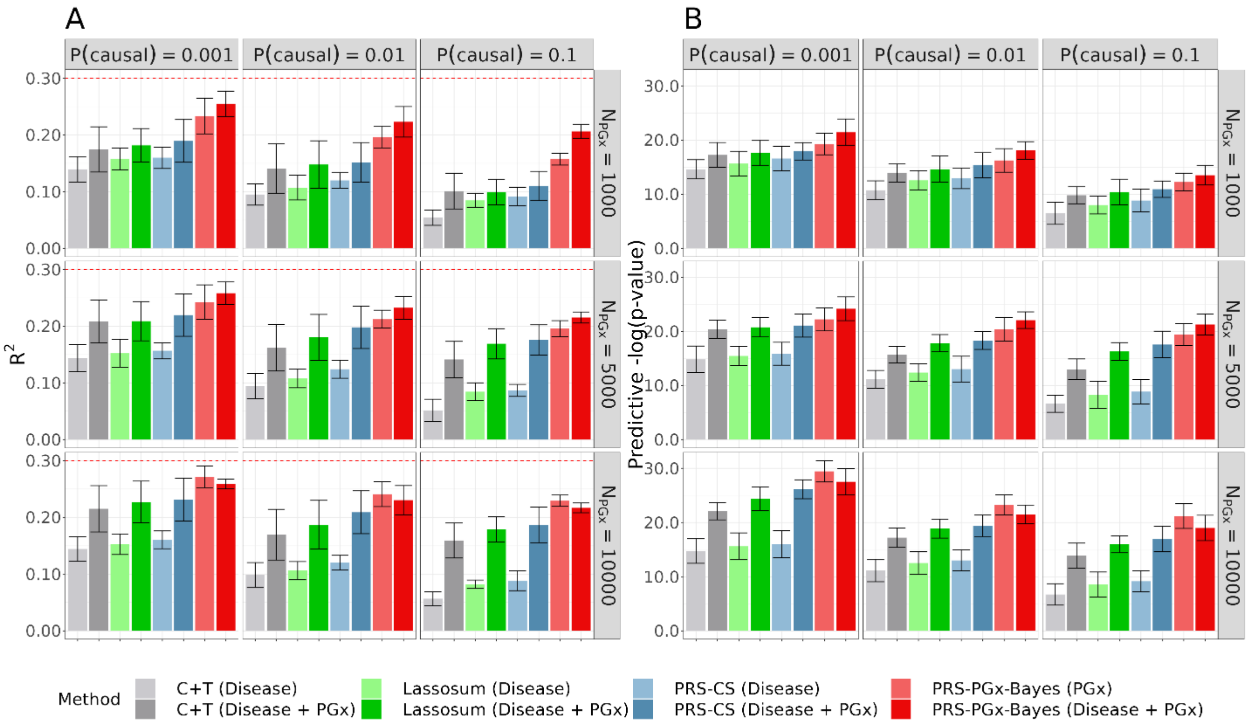

Figure S3: Methods comparison results (with external validation) among methods that use disease GWAS summary statistics only, PGx GWAS summary statistics only, or both. The prognostic and predictive effects were correlated, and in the same scale. Heritability was fixed at 0.3. The numbers of the causal variants for P(causal) = 0.001, 0.01 and 0.1 were 23, 226 and 2263, respectively. The PGx GWAS summary statistics in the base cohort were calculated with 1,000, 5,000, and 10,000 subjects, respectively; the disease GWAS summary statistics in the base cohort were calculated with 50,000 subjects. The tuning parameters were selected in an independent validation cohort. The performance was assessed in terms of (A) prediction accuracy R^2, and (B) predictive p-value for the two-sided PRS-by-treatment interaction test. Data were presented as mean values +/- standard deviations (error bars) with 10,000 replications, where results were calculated from the target cohort.

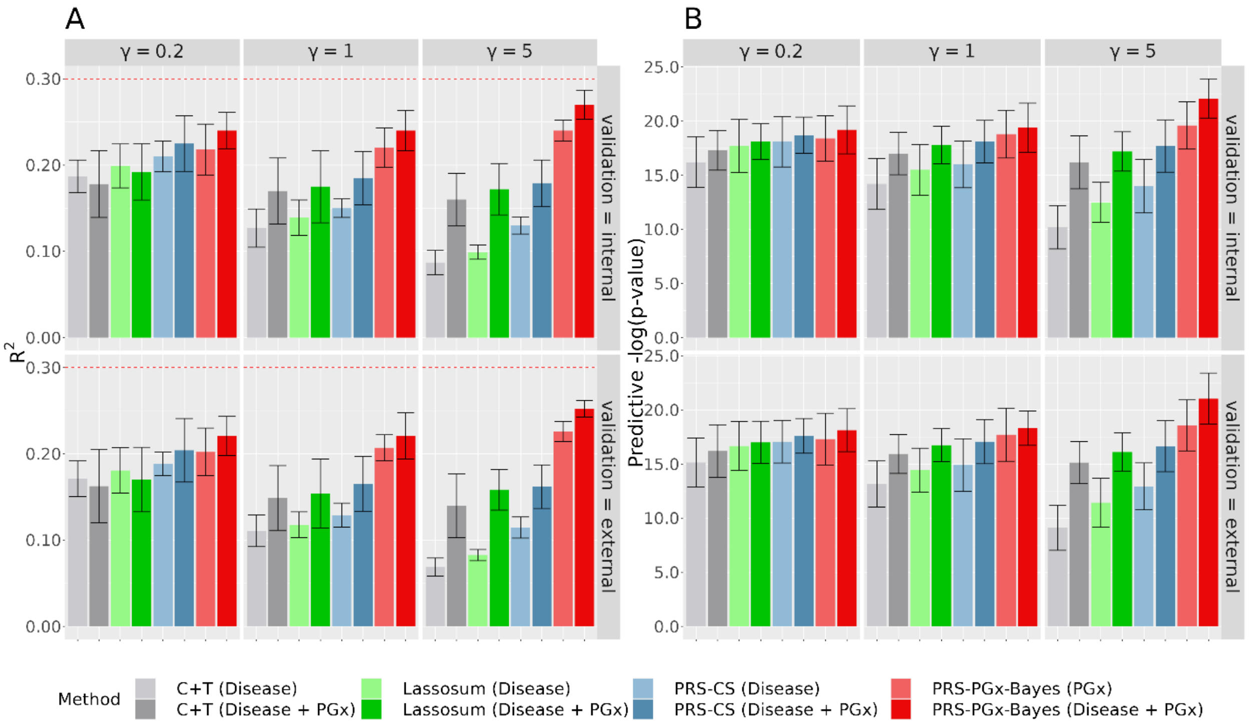

Figure S4: Methods comparison results (with either internal or external validation) among methods that use disease GWAS summary statistics only, PGx GWAS summary statistics only, or both. The prognostic and predictive effects were correlated, but in the different scales. When the predictive to prognostic effect size ratio $\gamma = 0.2$, the prognostic effect dominated the predictive effect; when the predictive to prognostic effect size ratio $\gamma = 5$, the predictive effect dominated the prognostic effect; when the predictive to prognostic effect size ratio $\gamma = 1$, the prognostic and predictive effects were in the same scale. Heritability was fixed at 0.3. The number of the causal variants for P(causal) = 0.01 were 226. The PGx GWAS summary statistics in the base cohort were calculated with 5,000 subjects; the disease GWAS summary statistics in the base cohort were calculated with 50,000 subjects. The performance was assessed in terms of (A) prediction accuracy R^2, and (B) predictive p-value for the two-sided PRS-by-treatment interaction test. Data were presented as mean values +/- standard deviations (error bars) with 10,000 replications, where results were calculated from the target cohort.

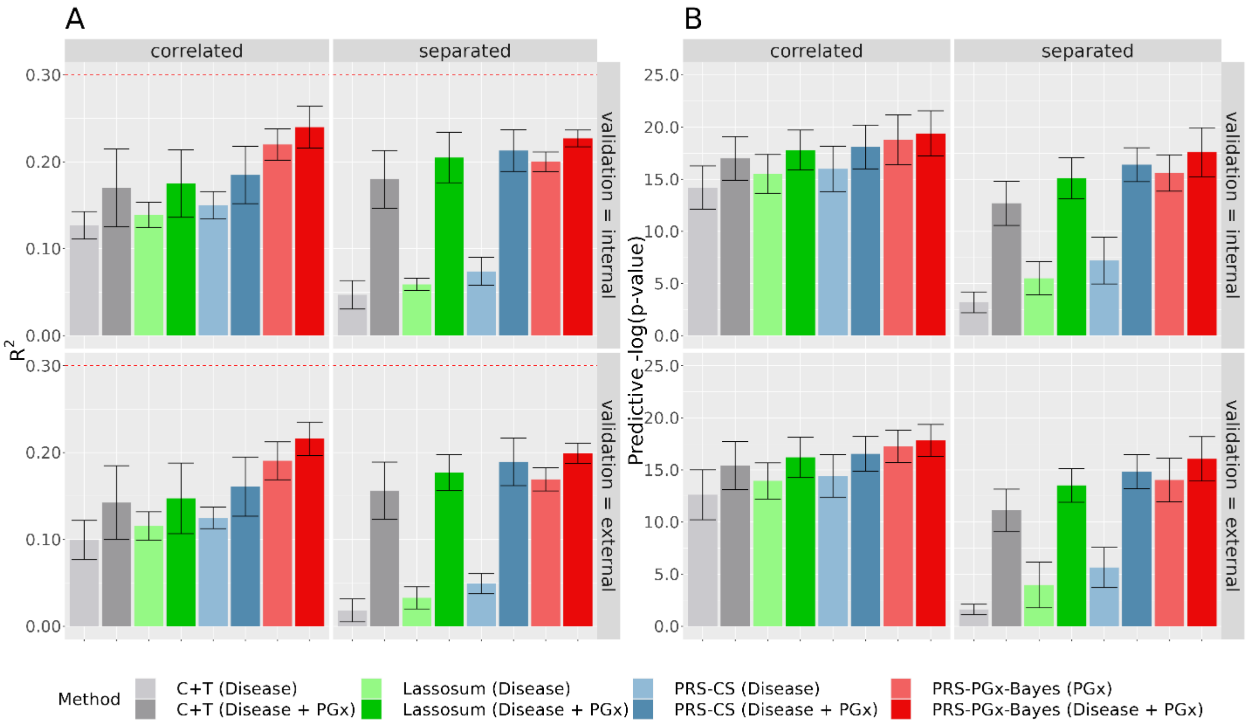

Figure S5: Methods comparison results (with either internal or external validation) among methods that use disease GWAS summary statistics only, PGx GWAS summary statistics only, or both. The prognostic and predictive effects were either correlated or fully separated, but in the same scale. Heritability was fixed at 0.3. The number of the causal variants for P(causal) = 0.01 were 226. The PGx GWAS summary statistics in the base cohort were calculated with 5,000 subjects; the disease GWAS summary statistics in the base cohort were calculated with 50,000 subjects. The performance was assessed in terms of (A) prediction accuracy R2, and (B) predictive p-value for the two-sided PRS-by-treatment interaction test. Data were presented as mean values +/- standard deviations (error bars) with 10,000 replications, where results were calculated from the target cohort.

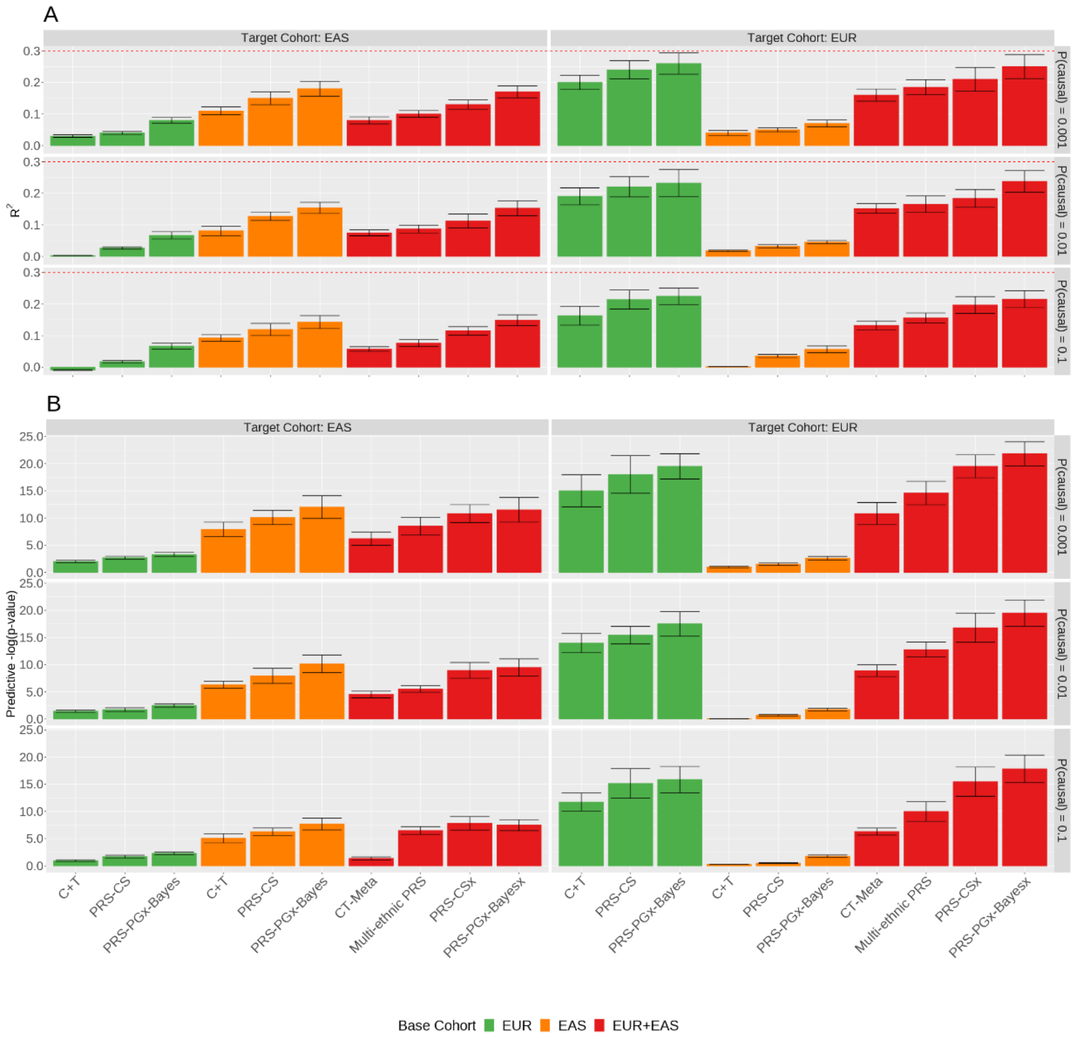

Figure S6: Methods comparisons between single-ethnic methods (using EUR or EAS alone) and trans-ethnic methods (using EUR and EAS jointly) when the effect correlation between EUR and EAS populations $\rho_P = 0.1$. The prognostic and predictive effects were correlated, and in the same scale. Heritability was fixed at 0.3. The numbers of the causal variants for P(causal) = 0.001, 0.01 and 0.1 were 12, 119 and 1188, respectively, for EUR population; and were 11, 106 and 1065, respectively, for EAS population. In the base cohort, the disease GWAS summary statistics of EUR and EAS populations were calculated based on 50,000 and 10,000 subjects, respectively; the PGx GWAS summary statistics of EUR and EAS populations were calculated based on 5,000 and 1,000 subjects, respectively. Sample sizes of EUR and EAS populations in the target cohort were 5,000 and 1,000, respectively. The tuning parameters were selected via cross-validation in the target cohort. The performance was assessed in terms of (A) prediction accuracy R2, and (B) predictive p-value for the two-sided PRS-by-treatment interaction test. Data were presented as mean values +/- standard deviations (error bars) with 10,000 replications, where results were calculated from the target cohort.

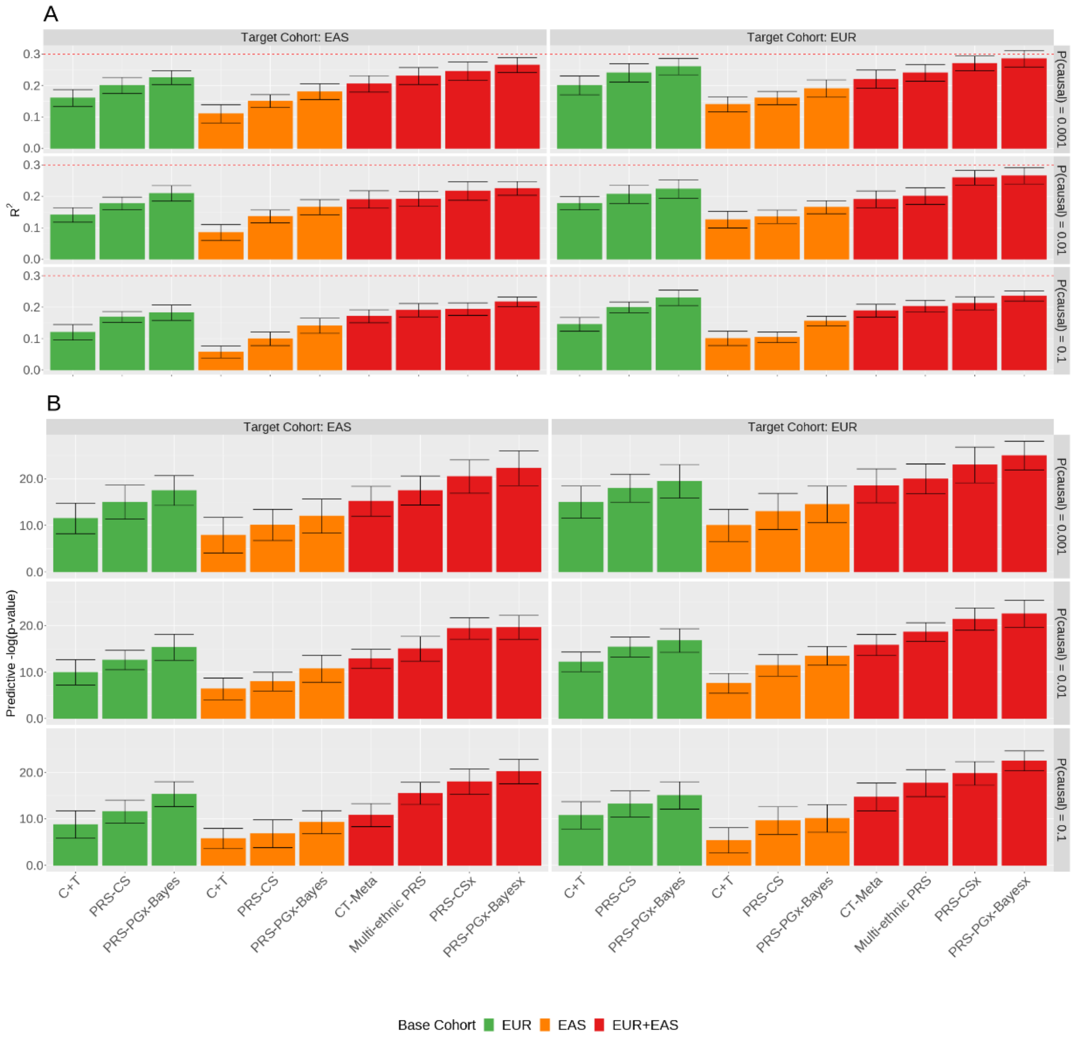

Figure S7: Methods comparisons between single-ethnic methods (using EUR or EAS alone) and trans-ethnic methods (using EUR and EAS jointly) when the effect correlation between EUR and EAS populations $\rho_P = 0.9$. The prognostic and predictive effects were correlated, and in the same scale. Heritability was fixed at 0.3. The numbers of the causal variants for P(causal) = 0.001, 0.01 and 0.1 were 12, 119 and 1188, respectively, for EUR population; and were 11, 106 and 1065, respectively, for EAS population. In the base cohort, the disease GWAS summary statistics of EUR and EAS populations were calculated based on 50,000 and 10,000 subjects, respectively; the PGx GWAS summary statistics of EUR and EAS populations were calculated based on 5,000 and 1,000 subjects, respectively. Sample sizes of EUR and EAS populations in the target cohort were 5,000 and 1,000, respectively. The tuning parameters were selected via cross-validation in the target cohort. The performance was assessed in terms of (A) prediction accuracy R2, and (B) predictive p-value for the two-sided PRS-by-treatment interaction test. Data were presented as mean values +/- standard deviations (error bars) with 10,000 replications, where results were calculated from the target cohort.

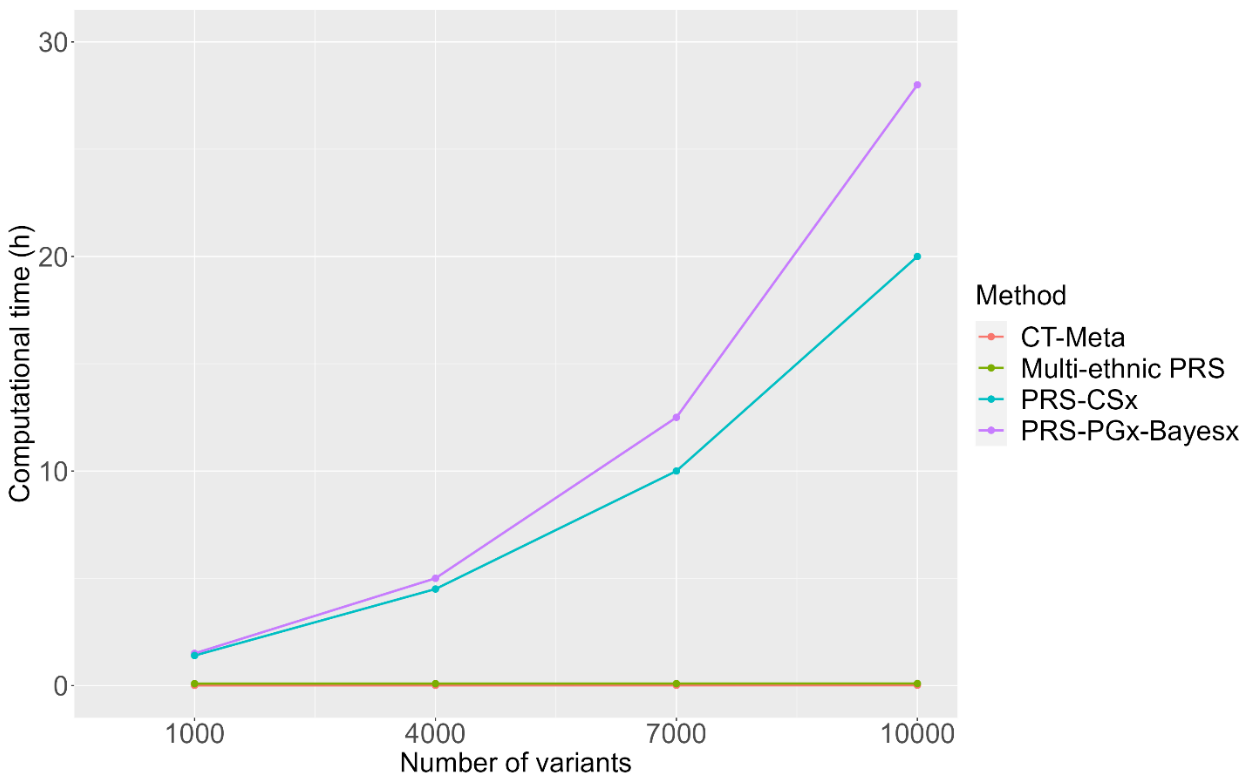

Figure S8: Computational time comparison between Bayesian based methods (PRS-PGx-Bayesx and PRS-CSx) and p-value thresholding based methods (CT-Meta and Multi-ethnic PRS). The computational time of PRS-PGx-Bayesx is based on 1,000 MCMC iterations. The number of variants = 1,000, 4,000, 7,000, and 10,000.

Supplementary Tables

Table S1. Summary of 90 identified papers following the review workflow in Figure S1.

Table S2. Summary of 23 existing methods and two newly proposed analysis strategy and method to construct PRS. The methods are categorized into four different types: Clumping and Thresholding (C+T), Machine Learning (ML), Best Linear Unbiased Prediction (BLUP), and Bayesian regression. The two new analysis strategy and method are PRS-PGx-Bayes (Disease + PGx) to leverage both disease and PGx GWAS summary statistics and PRS-PGx-Bayesx for trans-ethnic PRS analysis.

Table S3. Summary of existing mtPRS methods with pros, cons, and their performances in simulation studies from [2] and our additional simulation analyses (the detailed results are not shown). The methods are categorized into regression-based approaches, meta-/multi-GWAS-based approaches, BLUP-based approach, PCA-based approach, and omnibus (more robust) approach.

**Category**	**Method**	**Description**	**Pros**	**Cons**	**Performances in Simulation Studies**
Regression	mtPRS-MR	Combine multiple PRSs using multivariate regression by considering them as predictors	• Flexibly handle different effect correlation patterns (i.e., magnitudes, directions) between multiple traits in the base cohort and the drug response in the target cohort	• Handle correlations among traits implicitly<br>• Lack of clear biological interpretation	• Robust to different genetic architectures
	mtPRS-ML	Combine multiple PRSs using penalized regression by considering them as predictors			• Robust to different genetic architectures<br>• Generally with larger power than mtPRS-MR
Meta-GWAS	mtPRS-minP	Aggregate multiple GWAS summary statistics by selecting SNPs according to minimal p-value across traits	• Easy to implement<br>• Easy to interpret biologically	• An outlier from a trait may dominate the results	• Generally with lower power than other mtPRS methods, especially when the effect directions are different or the signal sparseness is high
	mtPRS-GSEM	Aggregate multiple GWAS summary statistics using a structural equation model	• Jointly analyze GWAS summary statistics to boost power<br>• More complicated model with larger power than minP		
BLUP	wMT-SBLUP	Combine single-trait predictors with BLUP properties in a weighted index calculated from SNP heritability, correlations between traits, and between the phenotype and BLUP predictors	• Incorporate both trait-trait correlation and trait-phenotype correlation	• Infeasible under PGx settings since wMT-SBLUP requires genetic correlations between traits in the base cohort and the drug response in the target cohort to determine weights, which are hard to obtain	• Robust to different genetic architectures<br>• Generally with larger power than regression-based methods
PCA	mtPRS-PCA	Combine multiple PRSs with weights calculated by performing PCA on genetic	• Easy to interpret biologically<br>• Handle genetic correlations among traits explicitly	• Do not consider different effect correlation patterns (i.e., magnitudes, directions) between multiple traits in the base cohort	• Outperform other mtPRS methods when traits are similarly correlated,

		correlation matrix among traits		and the drug response in the target cohort<br>• Rely on the correct estimation of genetic correlation matrix	with dense signal effects, and in similar effect directions
	mtPRS-O	Combine mtPRS-PCA, mtPRS-ML and all single-trait PRSs (stPRSs) using Cauchy Combination Test to detect the association	• Robust to different genetic architectures	• Only for the association test	• Robust and achieve optimally larger power compared with other mtPRS methods for association test

Supplementary References

1. Berisa T, Pickrell JK. Approximately independent linkage disequilibrium blocks in human populations. Bioinformatics. 2016;32:283.
2. Zhai S, Guo B, Wu B, et al. Integrating multiple traits for improving polygenic risk prediction in disease and pharmacogenomics GWAS. Briefings in Bioinformatics. 2023;24(4):bbad181.
